# Acetylation of Eugenol over 12-Molybdophosphoric Acid Anchored in Mesoporous Silicate Support Synthesized from *Flint* Kaolin

**DOI:** 10.3390/ma12182995

**Published:** 2019-09-16

**Authors:** Alex de Nazaré de Oliveira, Erika Tallyta Leite Lima, Deborah Terra de Oliveira, Rômulo Simões Angélica, Eloisa Helena de Aguiar Andrade, Geraldo Narciso da Rocha Filho, Carlos Emmerson Ferreira da Costa, Fabiola Fernandes Costa, Rafael Luque, Luís Adriano Santos do Nascimento

**Affiliations:** 1 Graduation Program in Chemistry, Federal University of Pará, Augusto Corrêa Street, Guamá, Belém PA 66075-110, Brazil; 2 Laboratory of Oils of the Amazon, Federal University of Pará, Perimetral Avenue, Guamá, Belém PA 66075-750, Brazil; 3 Department of Exact and Technologic Sciences, Federal University of Amapá, Rod. Juscelino Kubitschek, km 02-Jardim Marco Zero, Macapá AP 68903-419, Brazil; 4 Graduation Program in Biotechnology, Federal University of Pará, Augusto Corrêa Street, Guamá, Belém PA 66075-110, Brazil; 5 Laboratory of X-Ray Diffraction, Federal University of Pará, Augusto Corrêa Street, Guamá, Belém PA 66075-110, Brazil; 6 Adolpho Ducke Laboratory, Botany Coordinating, Museu Paraense Emílio Goeldi, Perimetral Avenue, Terra Firme, Belém PA 66077-830, Brazil; 7 Campus of Salinópolis, Federal University of Pará, Salinópolis, Pará CEP 68721-000, Brazil; 8 Faculty of Sciences, Research Institute of Chemistry, Peoples Friendship University of Russia (RUDN University), 6 Miklukho Maklaya str., 117198 Moscow, Russia; 9 Department of Organic Chemistry, Universidad de Córdoba, Ctra Nnal IV-A, Km 396, E14014 Cordoba, Spain

**Keywords:** acetylation, eugenyl acetate, 12-molybdophosphoric acid, *flint* kaolin, AlSiM, heterogeneous catalysis

## Abstract

A new prepared catalyst, 12-molybdophosphoric acid (HPMo) anchored to the mesoporous aluminosilicate AlSiM, synthesized from Amazon kaolin, was characterized and used as a heterogeneous acid catalyst for the production of eugenyl acetate by acetylation of eugenol with acetic anhydride. The effect of various reaction parameters, such as catalyst concentration, eugenol/acetic anhydride molar ratio, temperature and reaction time, was studied to optimize the conditions of maximum conversion of eugenol. The kinetics studies showed that in eugenol acetylation, the substrate concentration follows a first order kinetics. The results of activation energy was 19.96 kJ mol^−1^ for HPMo anchored to AlSiM. The reuse of the catalyst was also studied and there was no loss of catalytic activity after four cycles of use (from 99.9% in the first cycle to 90% in the fifth cycle was confirmed), and an excellent stability of the material was observed. Based on catalytic and kinetic studies, HPMo anchored to AlSiM is considered an excellent catalyst.

## 1. Introduction

Eugenol (4-allyl-2-methoxyphenol), a major component of clove oil, is widely used as a fragrance and flavoring agent in cosmetic, food and pharmacological products [1,2]. In addition, studies have shown that the therapeutic use of eugenol has great potential as antibiotic, anticarcinogenic, antimicrobial, antioxidant, anti-inflammatory, fungicidal, larvicidal and other agents [3,4,5,6,7,8]. However, in high concentrations, it has an adverse effect of provoking inflammatory and allergic reactions, possibly due to its low instability, which favors the formation of phenoxyl radicals via its pro-oxidant activity [2,9]. Despite the fact that its structure is polyfunctional and readily accessible, eugenol is considered as a useful starting material for producing valuable derivatives through chemical synthesis [2,10,11,12,13,14,15] or biochemistry [1,4,9,16,17,18].

Eugenol derivatives can be obtained by reactions on the hydroxyl group of its structure via acetylation [4,15,19] or benzoylation [2,9,20]. Investigations have shown that this modification also improves antioxidant and larvicidal activities 2 or 5 times, respectively, moreover to the reduced level of cytotoxicity compared to its precursor, eugenol [4,7]. Futhermore, studies have found that small amounts of eugenyl acetate are effective against the development of larvae of the *Aedes aegypti* mosquito [4,14,21]. The results reported in the literature ensure the potential application of eugenyl acetate in formulations of insecticides [4,7].

The *Aedes aegypti* mosquito is the virus vector of diseases such as dengue fever, yellow fever, Chikungunya, zika and cases of microcephaly in newborns [4,22]. For dengue disease, there are no drugs or vaccines available to prevent the spread of the virus so that the remaining method of controlling the propagation of its vector through the use of insecticides to prevent adult mosquito infestation or the use of larvicides synthetics such as organophosphates, organochlorines and others [6,22]. Mosquito population control is more effective in the larval stage than in adulthood [21]. The eugenyl acetate, besides being an efficient larvicide, still complies with the principles of green chemistry because it comes from biomass [4,6,7,14,21].

Most of the time the current chemical route to synthesize eugenol esters is quite problematic by making use of homogeneous catalysis [13,21,23,24], causing serious problems of reactor corrosion, volatile generation of unwanted effluents, impurity and unsatisfactory yield of the product, as well as a possible adverse impact on the environment, and in this sense the search for alternative heterogeneous catalysts has been constant [2,9,20]. Biocatalytic processes to obtain the ester, in spite of being alternative and environmentally friendly, in many cases still present high costs, mainly when immobilized, besides the low reuse capacity [1,10,16].

Thus, the use of heterogeneous catalysts is particularly advantageous because they are versatile, possess high operational efficiency under moderate reaction conditions, and additionally they provide a more ecological and economical route for esterification reactions [25,26,27,28,29,30,31]. In recent years, some acidic solids have been used as catalysts for the esterification or acetylation of eugenol, for example modified acid zirconia (UDCaT-5) [2] and ionic resins [10,11,12], which not only can be easily recovered and reused, but also favored the development of continuous production processes for a series of industrially important productions.

Heteropolyacids (HPAs), especially those of the Keggin series, are widely used as catalysts for the synthesis of fine and specialized chemicals [32,33,34,35,36]. Due to their strong acidity, they generally exhibit higher catalytic activities than conventional catalysts, such as mineral acids, ion exchange resins, mixed oxides, zeolites, etc. Also, HPA catalysis has no side reactions, such as sulfonation and chlorination, which often occur with mineral acids [34].

In the literature there are reports that the solid HPA, used as a homogeneous catalyst, and silica-supported HPAs acting as heterogeneous catalysts, are efficient for isomerization reactions of α-pinene oxides [34], limonene [32], cyclization of nerolidol and farnesol [36] and aldehyde cycloaddition [35]. However, studies on the use of heteropoly acids supported in mesoporous materials for the production of eugenyl acetate are not reported.

The nature of the support is an important factor for a successful reaction towards the best catalytic activity [37]. MCM-41 is the most studied member of the M41S family applied as catalytic support because of its regular structure, large surface area and uniform pore size, ranging from 15 to 100 Å [30,38,39,40,41]. The structure of the MCM-41 represents a hexagonal arrangement of cylindrical channels consisting of amorphous silica [41]. In the synthesis of MCM-41 the structure-directing agent cetyltrimethylammonium bromide (CTABr) and the tetraethylorthosilicate (TEOS) are the source of silica generally used. TEOS besides being toxic has a high cost [42,43]. Due to economic and environmental issues, research has been intensified by new sources of low-cost silica, such as metakaolin, for the synthesis of ordered mesoporous materials [44,45,46].

The Amazon *flint* kaolin (KF) is an industrial waste disposed of in the mine after the exploitation of soft kaolin, generating a considerable environmental impact, being constituted mainly by kaolinite clay, presenting high levels of silicon, aluminum and iron [25,47,48]. Our research group has aimed to add value to wasted kaolinite as alternative to minimize environmental impacts. Previous studies showed wasted kaolinite as feedstock for catalyst synthesis [25,26,29,31], catalytic supports [30,49] and for mesoporous aluminosilicate synthesis [27], for all cases, they were efficient for the esterification of free fatty acids, directed to the field of the production of biofuels [25,26,27,28,29,30,31]. The *flint* kaolin also shows potential as a starting material for zeolite synthesis [47].

In this sense, the objective of this study was to investigate an alternative to minimize problems caused by *flint* kaolin in mining, using this waste as a source of silica and aluminum in the synthesis of AlSiM. This work is the first report on the valorization of *flint* kaolin containing Al, Si, and a high content of Fe and Ti and other metals in small quantities for which pretreatment and purification steps [27] before the AlSiM synthesis.

In this work, also for the first time, the effect of AlSiM support on the catalytic activity of HPMo for the acetylation of eugenol was investigated. The support and catalysts synthesized were characterized by several physico-chemical techniques. The catalytic activity was evaluated for the production of eugenyl acetate by acetylation of eugenol. The influence of several reaction parameters (such as catalyst concentration, eugenol molar ratio/acetic anhydride, temperature and reaction time) in the catalytic performance was also studied. A kinetic study was performed and the order of reaction, reaction rate constant and apparent activation energy were determined as well as a catalyst recycling study was realized. As far as we know, no attempt to use HPA catalysts anchored in mesoporous silicates for this reaction has been made before.

## 2. Materials and Methods

### 2.1. Raw Material and Chemicals

12-molybdophosphoric acid (H_3_PMo_12_O_40_.H_2_O, HPMo, Mo = 63.08%, VETEC, Darmstadt, Germany), sulfuric acid (H_2_SO_4_, 98%, ISOFAR, Duque de Caxias, Rio de Janeiro, Brazil) and hydrochloric acid (HCl, 37%, Fmaia, Belo Horizonte, MG, Brazil), cetyltrimethylammonium bromide (C_16_H_33_(CH_3_), CTABr, Vetec, Rio de Janeiro, Brazil), ethanol (EtOH, 98%, Nuclear, São Paulo, SP–Brazil) and sodium hydroxide (NaOH, VETEC, Rio de Janeiro, Brazil), eugenol (EugOH, 99% Sigma Aldrich, Darmstadt, Germany) and acetic anhydride (AA, Nuclear, São Paulo, SP–Brazil). All chemical reagents and solvents were analytical grade and used without further purification. The *flint* kaolin was kindly provided by the UFPA Institute of Geology from the Capim River Region (Pará Brazil).

### 2.2. Preparation of Catalysts

#### 2.2.1. Thermal and Chemical Treatment of Amazon *Flint* Kaolin

**Scheme 1:**
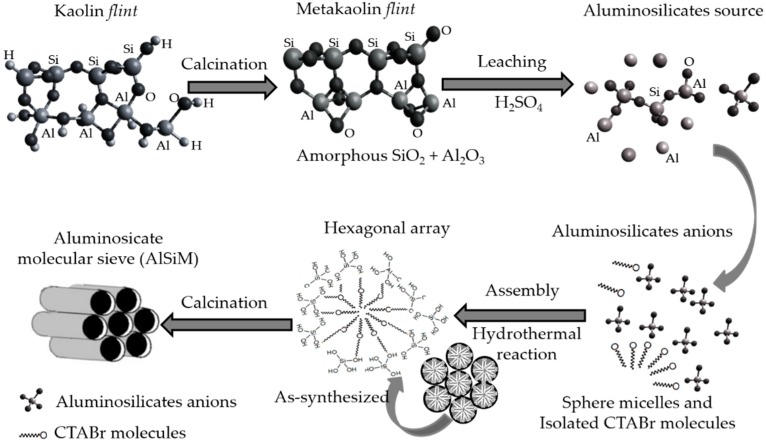
Schematic model for the possible formation of AlSiM from natural *flint* kaolin.

The *flint* kaolin was triturated, and the sandy fraction was separated for retention in a sieve. The fraction below 62 μm particle size was then collected, diluted in distilled water and centrifuged for separation of the silt fraction, thereby obtaining the clay fraction [25]. The *flint* kaolin was calcined at 750 °C for 5 h to give the metakaolinite (MF) phase which is amorphous. This material was then leached at 90 °C for 1 h with 2.5 mols L^−1^ H_2_SO_4_ solution in a MF/H_2_SO_4_ molar ratio of 1:10. After leaching, the product was filtered, washed with 100 mL of 0.5 mols L^−1^ H_2_SO_4_ solution and with water to neutral pH, and then dried at 120 °C for 12 h. The sample obtained after the acid leaching was denominated MFL (Scheme 1). All of these procedures are as described by Lima et al. [27].

#### 2.2.2. Synthesis of Mesoporous Aluminosilicate (AlSiM) by the Hydrothermal Method

The AlSiM synthesis procedure was based on literature methodology [27] where 3 g of the MFL was added in a solution composed of 1.8168 g of CTABr, 0.6172 g of NaOH and 134.7 mL of distilled water which was stirred for 24 h. After this period the solution was hydrothermally treated in a stainless steel autoclave-coated teflon vessel at 110 °C for 24 h. The formed hydrogel was filtered and washed with distilled water and dried at 110 °C for 12 h and calcined at 540 °C for 5 h for removal of the surfactant (CTABr). Based on the procedures described above, it was proposed a possible process for the preparation of ordered mesoporous AlSiM material from natural kaolin *flint* and is summarized in according to Scheme 1 adapted from literature [42,43,50].

#### 2.2.3. Catalyst Synthesis (HPMo Anchored to AlSiM)

**Scheme 2:**
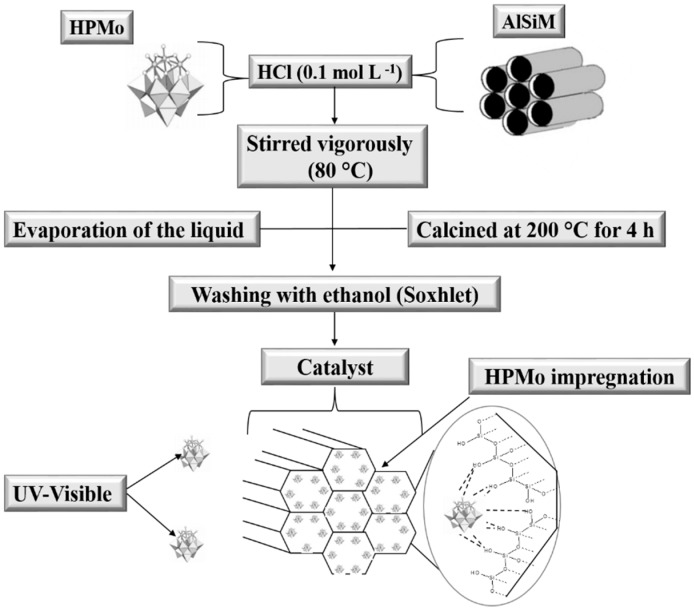
Procedure for synthesizing the HPMo catalyst anchored in AlSiM.

The HPMo anchored to the AlSiM was synthesized by impregnation according to Pires et al. [30] and Oliveira et al. [28]. Each gram of AlSiM was dispersed under vigorous stirring in a 0.1 mol L^−1^ aqueous HCl solution, which already contained the desired amounts of HPMo (0.11 g, 0.25 g and 0.43 g). The resulting mixture of HPMo and AlSiM was heated at 80 °C under constant stirring until complete evaporation of water. The solid obtained was pulverized and dried at 120 °C for 12 h and finally calcined at 200 °C for 4 h at a heating rate of 10 °C min^−1^. The samples obtained were designated as xHPMo/AlSiM, where x refers to the percentage of HPMo supported in the AlSiM, which varied in 10%, 20% and 30% by mass, according to Scheme 2 [28].

#### 2.2.4. Washing of AlSiM Anchored with HPMo

To eliminate excess HPMo on the surface of the material, which could contribute to homogeneous catalysis during the reaction, the xHPMo/AlSiM was subjected to a washing step. The xHPMo/AlSiM (3 g) was inserted into a Soxhlet extractor where it was washed with ethanol (300 mL) under constant reflux for 24 h, according to the procedure, adapted from the literature [28,51]. Thus, the measurement of HPMo in the washing solvent in the Soxhlet extractor also made it possible to indirectly estimate the amount of HPMo immobilized efficiently by ultraviolet-visible (UV-vis) spectroscopy [28,51]. At the end of this period, the solid was recovered by filtration, washed extensively with water at room temperature and posteriorly characterized.

### 2.3. Characterization

The final chemical composition of the calcined samples was determined using a Shimadzu EDX-700 energy dispersive X-ray spectrometer (EDX; EDX-700, SHIMADZU, Kyoto, Japan), with a rhodium X-ray source tube (40 kV, SHIMADZU, Kyoto, Japan). The spectra were obtained using 500 mg of the powder material, deposited in a bottom sample holder made of polyethylene film with low X-ray absorption, in the range of work.

X-ray diffractograms were obtained on a Bruker D8 Advance diffractometer (Bruker D8 Advance; Bruker Corp, Billerica, MA, EUA), using the powder method, at a 1° < 2θ <10° interval. Cu Kα (λ = 1.5406 Å, 40 kV e 40 mA) radiation was used. The 2θ scanning speed was 0.02° min^−1^. The distance (a_0_) between pore centers of the hexagonal structure was calculated by means of the equation a_0_ = 2*d*_100_/√3 [40].

N_2_ adsorption-desorption isotherms were obtained at liquid nitrogen temperature using a Micromeritics TriStar II model 3020 V1.03 apparatus (Micromeritics, Norcross, GA, USA). Before each measurement, the samples were outgassed at 200 °C for 2 h. The specific surface area (SSA) was determined according to the standard Brunauer-Emmett-Teller (BET) method. Pore diameter (D_p_) and pore volume (V_p_) were obtained by the Barret-Joyner-Halenda (BJH) method. The pore wall thickness (W_t_) was calculated according to Equation W_t_ = a_0_ − D_p_ [40].

Fourier transform infrared spectroscopy (FTIR) spectra were obtained from a spectrophotometer of Shimadzu (Kyoto, Japan) model IRPrestige-21A with a resolution of 32 and 100 scans and analyzed by Thermo Electron Corporation, IR 100 model with a resolution of 4 and 32 scans. For the analysis of all materials KBr pellets were used and the spectra were obtained in the region 4000–400 cm^−1^.

Diffuse ultraviolet-visible reflectance spectroscopy (DRS) were recorded, in the range of 200–500 nm, on a Shuimadzu UV-vis model ISR-2600 Plus spectrophotometer (EDX; EDX-700, SHIMADZU, Kyoto, Japan).

The Raman spectra were recorded using a Bruker, Vertex 70V spectrometer (Bruker D8 Advance; Bruker Corp, Billerica, MA, EUA), the system using a Diode Pumped Solid State (DPSS) laser by an Nd: YAG with an excitation laser equal to 514 nm. The detector is of germanium (Ge) kept under refrigeration of liquid nitrogen.

The thermogravimetric analysis (TGA/DTG) curves were obtained on a Shimadzu DTG-60H (Kyoto, Japan) model using N_2_ as purging gas (50 mL min^−1^). The analyses were performed from room temperature (~25 °C) to 900 °C at a rate of 10 °C min^−1^. About 10 mg of the samples were placed in platinum containers and heated from room temperature to 900 °C at a heating rate of 10 °C min^−1^ and using a N_2_ flow of 50 mL min^−1^.

The same technique was used for the quantification of the number of acid sites. For this, the samples prepared for evaluation of the presence of acidic sites in the infrared were submitted to thermal analysis and the TGA/DTG curves of the samples without adsorption and with adsorbed pyridine (Py) were evaluated. The number of acid sites was calculated from the difference in mass of the sample before and after being submitted to the adsorption of pyridine. The value of this difference corresponds to the mass of pyridine adsorbed where each mole of pyridine equals one mole of the acid site present on the surface of the catalyst. From these data the number of mmol of pyridine (nPy) per gram of sample was mathematically determined according to the method proposed by Nascimento et al. [25,29].

FTIR of adsorbed pyridine was the technique used to confirm the presence of Brønsted and Lewis acid centers in the catalysts [28,52]; 50 mg of sample was used for the acid sites identification that was made through previous heating at 120 °C during 90 min before the treatment with the pyridine probe molecule. After cooling, the sample were scaned within the range from 1700 and 1400 cm^−1^ in the FTIR spectrum [28,52].

The surface acidity was determined using acid-base titration [26,28,30]. In a typical measurement, 0.1 g of the solid was dispersed in 50 mL of 0.1 mol L^−1^ NaCl. The dispersion was stirred for 24 h and titrated with 0.1 mol L^−1^ NaOH in the presence of phenolphthalein.

Tests to determine the leaching of the HPMo of the catalysts were performed in UV-vis equipment of Thermo-scientific (Waltham, MA, USA), model Evolution array UV-vis spectrophotometer, with 200 to 600 nm scan and 30 scan resolution. The liquids were placed in a quartz tube. The calibration curve was constructed using equations (y = 0.1001x + 0.0032) of absorbance of λ_max_ = 310 nm with excellent correlation coefficient (R^2^ = 0.9999). To quantify HPMo leached in the reaction medium, another analytical curve was constructed from the post-reaction solution (1 to 5 mg L^−1^ of HPMo) which was appropriately diluted with 0.1 mol L^−1^ HCl to avoid any hydrolysis of the anion [PMo_12_O_40_]^3−^, with an equation (y = 0.0999 + 0.0025) absorbance of λ_max_ = 310 nm and an excellent correlation coefficient (R^2^ = 0.9999) based on that described in the literature [28,51].

### 2.4. Catalytic Tests

#### Acetylation of Eugenol

**Scheme 3:**
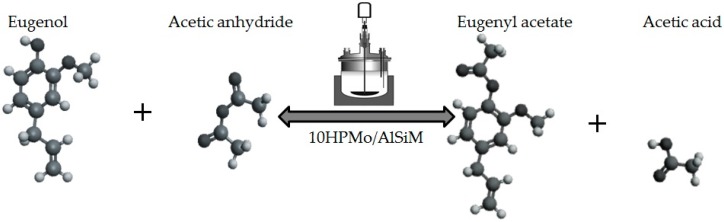
Design of eugenol esterification tests and acetic anhydride during catalytic tests.

Prior to the experiments, the catalysts were activated at 130 °C for 2 h. Tests of the catalysts were conducted in one run on a multirreator PARR 4871 (Parr Instrument Company, Moline, IL, USA). In a typical experiment, the eugenol was mixed with acetic anhydride at the molar ratio of 1:5 (Eugenol: AA) and 2% *w*/*w* of the solid acid catalyst (related to the Eugenol weight). The reaction mixture was stirred (500 rpm) and warmed from room temperature to 80 °C. Once the desired temperature was reached, the system was maintained for 40 min (Scheme 3). This period was considered as the kinetic contact time. At the end of the reaction, the reaction mixture was separated by centrifugation at 6000 rpm for 10 min. The separated catalyst by centrifugation was washed with ethanol to remove the polar and non-polar organic compounds adsorbed on the catalyst surface and oven dried at 150 °C for 4 h before being reused in the next cycle with fresh reagents to study their reusability. The recycle study was conducted in a manner similar to the above, but the mass of the catalyst and the other reactants were always recalculated prior to use in the subsequent reaction cycles under the best reaction conditions (80 °C for 40 min).

The acetic anhydride and acetic acid present in the filtrate were removed by rotevaporation at 70 °C for 4 h. After this procedure, an aliquot (1 μL) of the product was diluted in 1.5 mL of dichloromethane and analyzed by gas chromatography. Qualitative and quantitative analysis was performed through gas chromatography-mass spectrometry (GC-MS, Shimadzu QP2010 plus instrument, Shimadzu Corporation, Kyoto, Japan) under the following conditions: silica capillary column Rtx-5MS (30 m × 0.25 mm × 0.25 mm film thickness, Shimadzu Corporation, Kyoto, Japan); programmed temperature, 60–240 °C (3 °C min^−1^); temperature of the injector, 200 °C; carrier gas, helium, adjusted at a linear velocity of 1.2 mL min^−1^; type of injection, without division; the divided stream was adjusted to yield a ratio of 20:1; septal scan was a constant 10 mL min^−1^; EIMS (Electron Ionization Mass Spectra), electronic energy, 70 eV; temperature of the source of ions and bonding parts, 200 °C. The retention index was calculated for all volatile constituents using a homologous series of n-alkanes (C8–C32, Sigma-Aldrich, Saint Louis Missouri, EUA) The constituents were identified by comparing their mass spectra and retention indices (RI) with those in the system library (NIST-11, FFNSC-2) [53] and in the literature [54,55].

## 3. Results and Discussion

### 3.1. X-Ray Fluorescence (XRF)

The results of X-ray fluorescence (XRF) are shown in Table 1, where the increase of the Si/Al ratio throughout the process for the synthesis of the AlSiM material. The knowledge of the chemical composition of the silica precursor is fundamental because the percentage of silicon influences the stoichiometric calculations of the reagents necessary for AlSiM formation. XRF results of KF, MF and MFL used as a silica source are presented in Table 1. KF is constituted mainly by kaolinite (K). The high content of Fe_2_O_3_ and TiO_2_ may be related to the presence of accessory minerals such as hematite and anatase, and may be related to possible isomorphic substitutions of Al^3+^ with Fe^3+^ in the octahedral layer of this KF [25,47,48]. The results of XRF show that after the acid treatment of MF, there was a percentage reduction of about 88% of Al_2_O_3_ (37.92% to 4.74%) and 45% of Fe_2_O_3_ (2.78% to 1.62%), consequently increasing the amount of SiO_2_ in the materials, MFL (73.98%) and AlSiM (86.72%) [27].

The elemental analysis of the mesoporous materials (10, 20, 30) HPMo/AlSiM were carried out after calcination and washing with ethanol, in order to determine the actual loads of each prepared material. Calcination increases the incorporation of the catalyst (HPMo) in the support, transforming it into crystalline phase on the surface of the support that makes it insoluble in the reaction medium [28,56]. This confirmed the presence of Mo in the samples even after washing with ethanol. XRF results (Table 1) also demonstrated that the chemical compositions obtained, i.e., the Si/Mo molar ratio, are not the same as those expected after wet anchor synthesis (nominal molar ratio). After washing, there was an increase in the values of the Si/Mo ratio. These results confirm that essentially not all of the HPMo species present in the initial synthesis solution are anchored in the AlSiM structure. Observations such as these were previously reported in the literature [28,30,51,57]. The actual HPMo loads of the (10, 20, 30) HPMo/AlSiM samples were also evaluated using MoO_3_ analysis by XRF. Even with the difference between the nominal ratio and the measured mass being greater than 15% in all cases, the solids were referred to by their nominal compositions.

**Figure 1:**
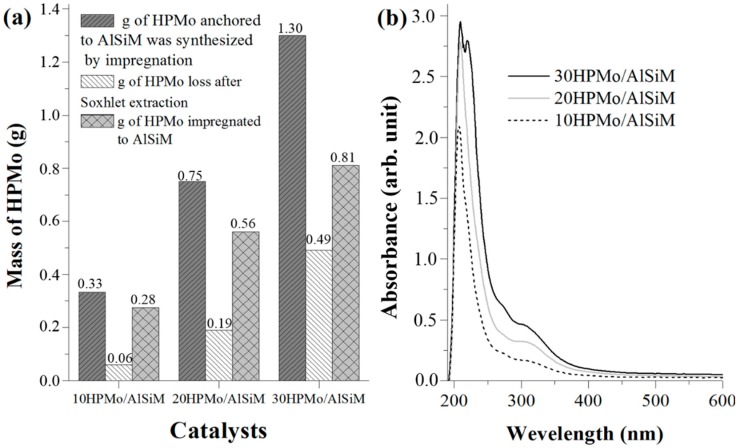
Quantification of HPMo losses by ultraviolet-visible (UV-vis) spectroscopy and calculation of immobilized HPMo (**a**); UV-vis spectra of the HPMo leached after the ethanolic washing of the supported materials (**b**); in all cases 3 g of AlSiM were used during the anchoring of HPMo.

UV spectroscopy analysis of the (10, 20, 30) HPMo/AlSiM sample washout supernatant (Scheme 2) provided an estimate of the amounts of HPMo not incorporated into the AlSiM (Figure 1a). The HPMo amounts incorporated into the matrices can be calculated by subtracting the initial amount of added HPMo minus the amount of HPMo detected by UV–vis in the supernatant [28,51].

The results are shown in Figure 1a, after 24 h of washing, the 10HPMo/AlSiM, 20HPMo/AlSiM and 30HPMo/AlSiM catalysts were found to have partial HPH losses near 15%, 25% and 38% in that order, supposedly mass has been leached from the initial amount. Therefore, the mass fraction of the actual HPMo immobilized on the final solid catalysts were recalculated and fell in the range of 8.5%, 16% and 21% by weight respectively. Based on the data presented on Table 1, it was found that 10HPMo/AlSiM is the most stable catalyst for leaching, losing about 15% of [PMo_12_O_40_]^3−^ during washing.

### 3.2. X-ray Powder Diffraction (XRD) Analysis

**Figure 2:**
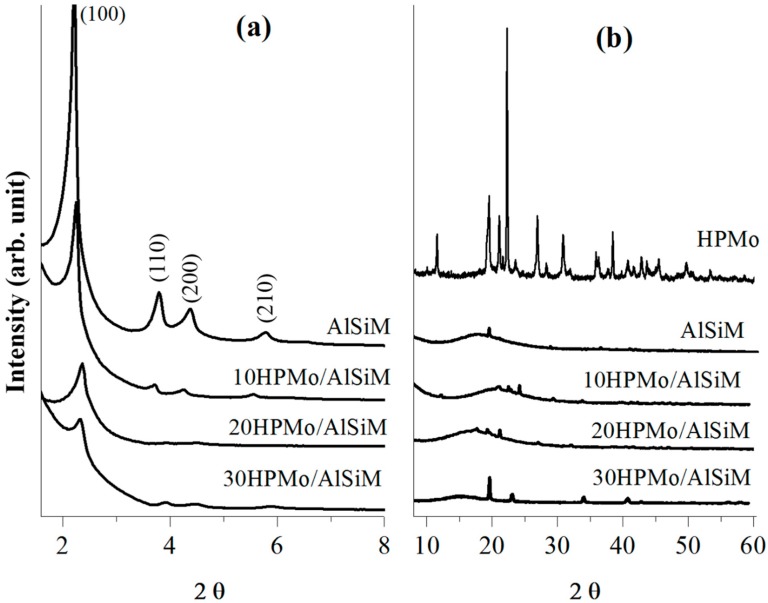
X-ray powder diffraction (XRD) pattern of AlSiM (calcined) and (10, 20, 30) HPMo/AlSiM samples. (**a**): Low angle, (**b**): High angle.

The diffraction patterns of the samples are shown in Figure 2. The AlSiM X-ray powder diffraction (XRD) pattern (Figure 2a) shows a very strong peak (100) at about 2.1° (2θ) and three weak peaks corresponds to the reflections (110), (200) and (210), suggesting a high ordering of the hexagonal pore arrangement in the material [44,45,50]. Possibly, the high Si/Al molar ratio of 23 (AlSiM, Table 1) favors the formation of the well defined mesoporous structure with intense peaks. This observation is in accordance with previously published data that proves the well ordered formation with molar relation Si/Al de 13.8 [58] e Si/Al de 29.3 [50].

Characteristic peaks of the hexagonal system were also found for the samples (10, 20, 30) HPMo/AlSiM. The anchoring of HPMo to AlSiM during synthesis of the (10, 20, 30) HPMo/AlSiM series) produces a deterioration of textural characteristics which is most significant at high HPMo (lower Si/Mo ratio, Table 1). With increasing HPMo content peak d_100_ reduced in intensity and the three reflections at angles greater than d_100_ (2θ = 3–6°) became larger and less intense in the samples. Four indexed peaks are observed at the diffractogram of the 10HPMo/AlSiM sample, and these peaks are atributted to the (100), (110), (200) e (210) reflections, typical of AlSiM matrix, while in the diffractograms of samples with 20% and 30% HPMo load, only the peak indexed to reflection (100) is clearly observed.

The partial disappearance of the upper peaks suggested that the (10, 20 and 30) HPMo/AlSiM1 samples had a less ordered structure compared to AlSiM1, but the mesoporous structure and hexagonal ordering were maintained after the introduction of HPMo [30,59,60,61]. These results show that the impregnation of HPMo caused a decrease of d_100_ and a_0_ and an increase of W_t_, (Table 2), reported in the literature indicating that HPMo may be well dispersed on the surface and inside the AlSiM pores, forming thin layers [60,62]. A summary of the values of d_100_ are presented in Table 2 along with the corresponding unit cell parameters (a_0_) and wall thickness (W_t_) of all samples.

XRD analysis of (10, 20, 30) HPMo/AlSiM materials were also extended to values greater than 2θ (range 10–60°) in order to obtain more information on the location and nature of HPMo species, or presence or absence of peaks attributed to HPMo (Figure 2b). Therefore, no prominent HPMo diffraction peaks were found in the XRD patterns of the supported samples as compared to pure HPMo XRD, which is indicative of the high dispersion of HPMo in the possibly monolayer carrier within the hexagonal channels of AlSiM [60,61], since the excess HPMo is eliminated by washing with ethanol. Peaks, observed in the samples, near 2θ = 21, 25 and 36° can be related to the minerals quartz (SiO_2_) anatase (TiO_2_) and hematite (Fe_2_O_3_) very common in *flint* kaolin (Table 1) of the Capim River Region [25,26,47].

### 3.3. Textural Properties

Calcination, dehydroxylation of kaolinite to the formation of metakaolinite can promote the behavior of leaching of clayey, increasing considerably the surface area and volume of pores (Table 2). The KF and MF showed small SSA (8 and 9 m^2^ g^−1^) with similar Vp (0.05 cm^3^ g^−1^) (Table 2). After calcination, the MF submitted to acid leaching presented higher SSA and V_p_, approximately 43 times and 11 times more than the starting MF, respectively.

**Figure 3:**
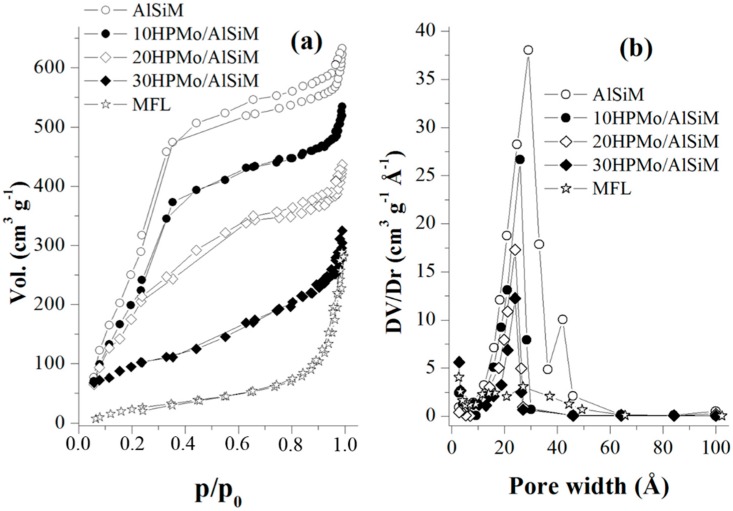
Nitrogen physisorption isotherms (**a**) and pore distribution. (**b**) of samples AlSiM and (10, 20, 30) HPMo/AlSiM.

The N_2_ physisorption isotherms and the pore size distribution curves of the MFL, AlSiM and (10, 20, 30) HPMo/AlSiM are shown in Figure 3a,b respectively. The MFL isotherm according to Du and Yang [50] is a mixture of types II and IV, with an initial portion similar to the type I isotherm and a H3 and H4 hysteresis mixture, indicative of narrow, slit-shaped pores.

The N_2_ physisorption isotherms and pore distribution (Figure 3a,b) of the AlSiM and (10, 20, 30) HPMo/AlSiM samples were classified as type IV according to the IUPAC classification, indicating the formation of structures mesoporous [40]. With a hysteresis cycle characteristic of uniform mesoporous material for AlSiM sample, the mesoporous materials obtained from metakaolin [44,46,50].

The isotherm of the AlSiM sample presented three more evident steps: first stage presents a relatively low pressure (p/p_0_ < 0.2) is explained by a monolayer adsorption of nitrogen in the walls of mesoporos for all the samples [46,50]. The presence of a pitch between 0.2 < p/p_0_ > 0.3 shows that the isotherms exhibit a marked inflection, characteristic of nitrogen capillary condensation in the hexagonally ordered cylindrical mesopores of materials that have a very uniform pore distribution [46,50], with a narrow hysteresis cycle [44]. The third phase at higher relative pressures (p/p_0_ > 0.34), a slight increase in the amount of N_2_ adsorbed, plateau region, was observed due to the multilayer adsorption on the external surface of the particles [46]. The increase in the neighborhood of p/p_0_ around 1.0 can be attributed to the presence of macropores corresponding to the spaces formed by the agglomeration of the mesoporous particles [46,50].

The AlSiM pore size distribution with Si/Al = 23 (Table 1), calculated by the BJH method, shows a sharp peak at about 2.8 nm, mean pore size at about 3.9 nm with a range of 15–50 Å in diameter, confirmed by the pore size distribution curve (Figure 3b). Thus, the pores formed in the mesoporous AlSiM are uniform and this result corroborates with the crystallinity observed in the XRD standard (Figure 2). An ordered mesoporous material with SSA (1071 m^2^ g^−1^) e V_p_ (1.05 cm^3^ g^−1^) was obtained by using the leached sample from KF as source of Si and Al. The results indicated that the porous properties of the mesoporous material obtained are very similar or even greater than those of the samples prepared by general methods using commercial kaolin [44,45,46,50] or synthetic silica [30,37,38,40,60,61,63].

The textural properties, such as surface area, pore diameter and pore volume, derived from N_2_ physisorption measurements are included in Table 2. The results show that the synthesized mesoporous aluminosilicate molecular sieve has a much higher surface area than the starting material. A comparison was made with mesoporous material from several literature reviews (Table 3). The results indicate that the sample synthesized in this study has better textural properties and within the required standards. This proves the viability of KF as a precursor of silicate in AlSiM synthesis.

As for the volume of adsorbed N_2_, it decreased with the increase of the HPMo content due to the disorder of the mesoporous structure of the samples. The adsorption isotherm form of 10HPMo/AlSiM with low HPMo content (Si/Mo = 24, Table 1) is similar to that of pure AlSiM. However, with high HPMo load (Si/Mo = 8, Table 1), the sample 30HPMoAlSiM, showed hysteresis absence, (Figure 3a). The anchoring of HPMo 30% on the AlSiM sample should be causing a reduction on the SSA (of 1071 to 728 m^2^ g^−1^), V_p_ (of 1.05 to 0.57 cm^3^ g^−1^) leading to higher values of wall thickness (of 1.17 to 1.39 nm) while the D_p_ fell from 3.85 to 3.21 nm, and the hysteresis absence suggest the partial blocking of the pores in this sample [64]. Thus, it can be assumed that most HPMo units, with a diameter close to 1.2 nm, are deposited within the AlSiM pore channels [60,61]. This conclusion is in agreement with data previously published in which low HPMo load (between 12% and 16%) showed high dispersion in mesoporous silica catalysts [59,60,61].

However, the pore size distribution curves of the HPMo/AlSiM samples (10, 20, 30) are in the range of 15–30 Å, with a narrow distribution centered, with maximums recorded around 2.4 nm, and with an increase in HPMo loads the height of the curves probably decreased as a consequence of AlSiM pore blockade (Figure 3b) [60,61,65]. The least wide pore size distribution of the HPMo/AlSiM samples (10, 20, 30) are related to their less disordered mesostructures, as shown by the XRD standard (Figure 2a) and this is reflected in the less accentuated slope of the second stage of the isotherm values of 0.3 < p/p_0_ > 0.4 (Figure 3a) compared to the AlSiM [64]. The data obtained from the adsorption and desorption branches of N_2_ are in agreement with others already published for the mesoporous materials anchored with HPMo [59,60,61,62,65,66,67].

### 3.4. Diffuse Reflectance Spectroscopy (DRS)

**Figure 4:**
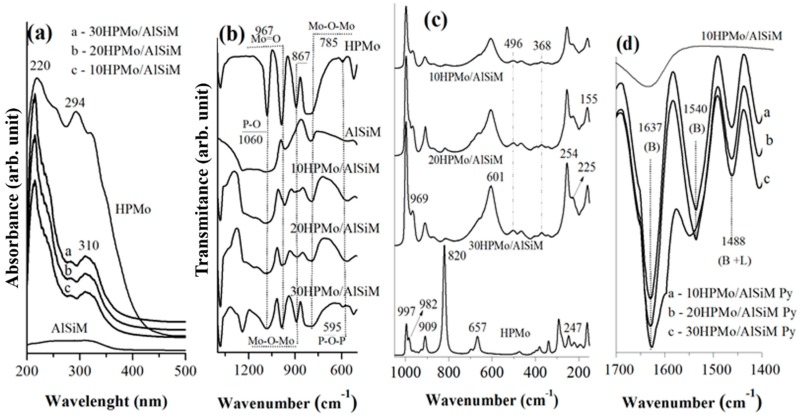
Diffuse reflectance spectroscopy (DRS) spectra of HPMo, AlSiM and (10, 20 and 30) HPMo/AlSiM samples (**a**); Fourier transform infrared spectroscopy (FTIR) spectra of the HPMo, AlSiM and (10, 20 and 30) HPMo/AlSiM (**b**) samples; Raman spectra of HPMo and (10, 20 and 30) HPMo/AlSiM (**c**); FTIR spectra of pyridine adsorption of samples (10, 20 and 30) HPMo/AlSiM (**d**).

Diffuse reflectance spectroscopy (DRS) is known to be a very sensitive probe for the identification and characterization of the coordination of metallic ions and their existence in the structure or extra position of structure of metal-containing mesoporous materials [38,40]. The UV-vis DRS spectra of the AlSiM, HPMo and (10, 20 and 30) HPMo/AlSiM samples are shown in Figure 4a. The HPMo DRS spectrum exhibits four absorption bands of 220, 294 and 310 nm, which are in line with that reported in the literature [68]. These bands can be attributed to metal bonding charge transfer (LMCT) (O^2−^ → M^6+^), which can be related to the metal with tetrahedral coordination (220 nm) and octahedral (310 nm) [38,40,61,68,69].

The DRS spectra of (10, 20, 30) HPMo/AlSiM (Figure 4a) have contributions of absorption bands centered at 220 and 310 nm, and these observations are in agreement with the literature [61,68,69,70]. These results may indicate that the metal was anchored in the AlSiM structure, and presumably the peak near 220 nm was attributed to the transfer of electrons from oxygen to the metal (O^2−^ → Mo^6+^), involving isolated atoms of Mo in tetrahedral coordination shifted to higher energies, characteristic of heteropolianions [PMo_12_O_40_]^3−^ [68,69]. The shoulder at 310 nm was associated with octahedrally coordinated, isolated Mo species [61,68,69,70].

Generally, the DRS profiles for the hybrid catalysts were similar to the corresponding mesoporous supports. This may be due to the formation of intermolecular bonding between silane groups (Si–OH) present on the AlSiM surface and HPMo, allowing the possible formation of tetracoordinated Mo ions, consisting of two Mo–O–Si, Si–O–Mo–O and two terminal connections Mo=O [59,63,70]. Thus, DRS data confirm the aforementioned results based on the analysis of FTIR and Raman spectra (Figure 4b,c) indicating the presence of chemical interactions between HPMo and AlSiM. Similar behaviors were previously reported for HPMo deposited in MCM-48 [70] and MCM-41 [61]. The AlSiM sample prepared from MF, on the other hand, shows a clear absorption near 330 nm typical of anatase [64]. The appearance of anatase signals in the DRS spectrum is corroborated by the XRF technique (Table 1) and in the sample (AlSiM) it is common to detect the anatase phase (TiO_2_) as it is an accessory mineral of the KF as well as the hematite [25,26,29,47,48].

From the characterization results presented above, it is possible to conclude that the incorporation method of the post-synthetic HPMo tested in this study allows to obtain a mesoporous AlSiM type material with 10% HPMo load and without considerable degradation of the initial AlSiM structure (Figure 2a). In fact, no prominent HPMo diffraction peaks were found in the XRD patterns of the (10, 20 and 30) HPMo/AlSiM samples, indicating high dispersion of HPMo (Figure 2b). This conclusion is in agreement with previously published data that proves the high dispersion of HPMo in mesoporous silicas [59,60,61].

### 3.5. Fourier Transform Infrared Spectroscopy (FTIR)

HPMo, AlSiM and (10, 20 and 30) HPMo/AlSiM FTIR spectra are shown in Figure 4b. In the AlSiM FTIR spectrum, there is a broad band between 1247–1060 cm^−1^ which can be attributed to asymmetric O–Si–O binding stretching vibrations, and a peak 967 cm^−1^ is assigned asymmetric stretching vibrations of terminal silanol groups (Si–OH) or aluminum (Si–O–Al) [46,50]. The vibration bands observed in the 790 and 460 cm^−1^ regions are attributed to the symmetrical and asymmetric bending of tetrahedral O–Si–O bonds, respectively [71], which are also slightly shifted to higher frequencies after calcination of the material [46,50]. The main characteristic bands of the Keggin structure in 1060 cm^−1^ (P–O), 967 cm^−1^ (Mo–O), 867 e 785 cm^−1^ (Mo–O–Mo) are observed for bulk HPMo material in consistency with previous reports [28,60,61,62,65,66]. The above vibrations are also visible on supported HPMo catalysts of less than 30 wt % and their corresponding intensities increase with increasing HPMo content, which indirectly proves that there are interactions between the silicon species and HPMo in the AlSiM framework hydrogen bonding [28,59,60,65]. Studies confirm that silane groups (967 cm^−1^) interact with HPMo species to form binding Si–O–Mo [37,60,61,62,66,72]. This observation is corroborated with the explanation reported by Khayoon and Hameed [59] in which the presence of silanol (Si–OH) groups in the silica porous wall contribute to react with HPMo to form Si–O–Mo–O bonds, which finally develop in thin layers on the inner walls of the surface pores of SBA-16. In addition, the crystalline phases of MoO_3_ and P_2_O_5_, which would be produced by the thermal decomposition (200 °C) of HPMo in samples (10, 20 and 30) HPMo/AlSiM, were not detected clearly in the spectral regions (1060 and 785 cm^−1^) possibly because they are partially overlapped by the vibration bands of the AlSiM structure. These results corroborate the fact that the primary structure of Keggin is preserved [60,61,65] after the synthesis procedure and calcination at 200 °C of the catalysts.

### 3.6. Raman Spectroscopy

The Raman technique is a useful characterization tool to observe the Keggin structures present in the AlSiM structure. The Raman spectra of the catalysts (10, 20 and 30) HPMo/AlSiM and HPMo by mass are given in Figure 4c. HPMo Raman spectrum shows Keggin structure characteristic bands at 997 cm^−1^ (Mo–O), 982, 909, 820 e 657 cm^−1^ (Mo–O–Mo) e 247 cm^−1^ (Mo–O) [65,70,73,74].

In the Raman spectra of the (10, 20, 30) HPMo/AlSiM materials, the characteristic bands of HPMo [70,73], appear gradually in 997, 969, 254 e 155 cm^−1^ (Figure 4c), which indicates the presence of a very strong interaction between the HPMo oxygen and the hydrogen of the AlSiM silanol group, which Raman is able to detect [64,65,75]. This confirms that HPMo retained in AlSiM, even after washing samples with ethanol [64,65,73]. Characteristic bands of the anatase (TiO_2_) appear gradually 155, 368, 496 e 609 cm^−1^, this confirms that TiO_2_ is a constituent of the AlSiM composition even at very low loading (Table 1), and the Raman technique was still able to detect it. Therefore, the presence of TiO_2_ incorporated in the AlSiM support favors the dispersion of HPMo, resulting in the disappearance of the 820 cm^−1^ band, but samples (10, 20, 30) HPMo/AlSiM [64]. The Raman spectra of the (10, 20, 30) HPMo/AlSiM samples are comparatively large, which may be due to the nature of the carrier [37]. These results are in line with the XRD standards (Figure 2b), which did not show HPMo crystalline peaks for these catalysts, regardless of the HPMo content.

### 3.7. Thermal Analysis (Thermogravimetric/Derivative Thermogravimetric (TG/DTG))

**Figure 5:**
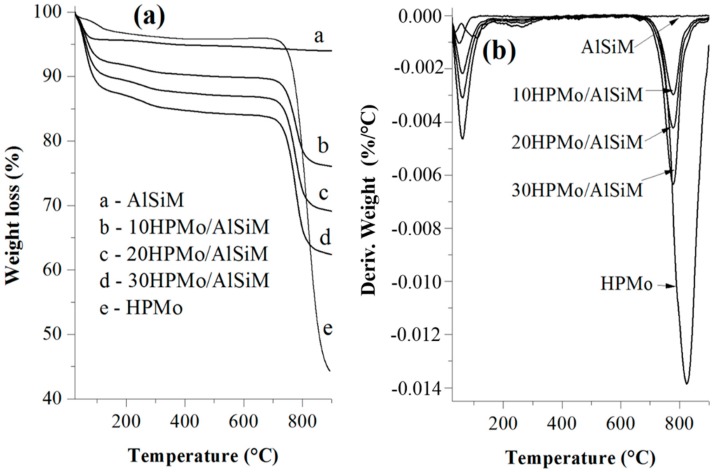
Thermogravimetric (TG) (**a**) and derivative thermogravimetric (DTG) (**b**) curves of the HPMo, AlSiM and (10, 20, 30) HPMo/AlSiM samples.

Thermogravimetric (TG) and derivative thermogravimetric (DTG) curves of the samples made are shown in Figure 5a,b respectively. Mass loss increases with HPMo loading and the sequence is 10HPMo/AlSiM (14.01%) < 20HPMo/AlSiM (21.05%) < 25HPMo/AlSiM (29.03%) < HPMo (58.19%). All the (10, 20, 30) HPMo/AlSiM catalysts and the HPMo by mass show a similar profile of mass loss in contrast to the relatively flat AlSiM (Figure 5a). This gradual mass loss is probably due to the inclusion of HPMo within the mesopores of AlSiM support.

DTG curves of the pure HPMo and the (10, 20, 30) HPMo/AlSiM samples exhibit endothermic peaks below 200 °C, corresponding to loss of crystallization or adsorbed water, forming the anhydrous heteropolioxo compound [28,65]. Previous studies have suggested that this loss of water involves catalyst deprotonation (HPMo) with simultaneous loss of oxygen from the network, but Keggin is primary unit is maintained [28,65]. In addition, weight changes and thermal effects were not observed at intermediate temperatures, suggesting the formation of anhydrous heteropoly anions [65]. However, a signal at 830 °C for HPMo and at 780 °C for (10, 20, 30) HPMo/AlSiM can be attributed to the crystallization of the oxides resulting from the total decomposition of Keggin units. All these results are in agreement with the literature [61,65].

Analyzing DTA curves (not shown), all the observed events were endothermic and can be attributed to the loss of adsorbed water and adsorbed gases (<200 °C) as well as loss of crystallization of the HPMo (>200 °C) present in these samples. No visible exothermic peak appears at 400 to 600 °C, suggesting strong interaction between HPMo and AlSiM. In contrast to bulk HPMo, the thermal stability of HPMo increases substantially after immobilization in AlSiM, which coincides with previous reports [61,65]. This may be due to the formation of intermolecular bonding between AlSiM and HPMo and indicated the presence of chemical interactions between them, behavior similar to that previously reported for HPMo deposited in mesoporous silicas [59,60,61,62,65,66,70].

The chemical analysis (Table 1) and FTIR vibration spectra with bands close to 1060, 867, 790 e 460 cm^−1^ (Figure 4a) confirm the existence of silane groups (Si–OH–Al, Si–OH–Fe) in the samples, even after the incorporation of HPMo [62,66]. The presence of HPMo in the prepared materials was also confirmed by FTIR as can be seen in Figure 4b. Bands characteristic of the Keggin type structure appeared around 1060, 967, 867 e 790 cm^−1^ and are characteristic of heteropolianions [PMo_12_O_40_]^3−^ [61,68,69,70]. In addition, data from chemical analyzes (Table 1) reveal the existence of species such as Fe, Al, Mo and P in the samples.

### 3.8. Surface Acidity

The surface acidity of AlSiM solids supported with (10, 20, 30) HPMo was evaluated by means of acid base titration [26,28,30] and using FTIR methodology and the TGA/DTG technique after adsorption of pyridine, and proved to be a good tool for the characterization of heterogeneous catalysts and quantification of acid sites, using pyridine as probe molecule [25,26,28,29].

The results of the acid analysis of the materials measured by base acid titration are presented in Table 3. The amount of active sites present in the samples were correlated with the hydroxyl consumption during the titration [26,28,30]. It was observed that all the supported catalysts have very high acidity in comparison to the support and that these values are directly proportional to the concentration of active species (MoO_3_) in AlSiM material. Analogously, the catalysts with higher HPMo loads showed higher surface acidity, which confirms the impregnation of HPMo [59].

**Figure 6:**
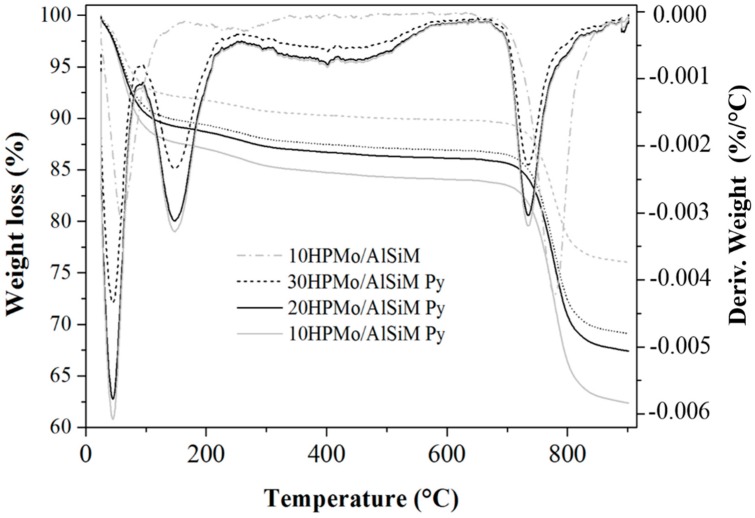
TGA/DTG curves of the catalysts without and with adsorbed pyridine: 10HPMo/AlSiM and (10, 20 and 30) HPMo/AlSiM Py; where Py are samples with pyridine.

In another study we decided to quantify the total amount of acid sites using the TG/DTG technique after the pyridine adsorption described by Nascimento et al. [25,29]. The TGA/DTG curves clearly indicated that samples with adsorbed pyridine, (10, 20, 30) HPMo/AlSiM Py, lost more mass than the samples without adsorbed pyridine, 10HPMo/AlSiM, as can be seen in Figure 6 The TGA/DTG curves of the adsorbed pyridine samples show a loss between 150 and 250 °C which can be attributed to both the water and the adsorbed pyridine, and a continuous loss above 250 °C attributed to the loss of chemically adsorbed pyridine [25,26,28,29]. Therefore, through the methodology described by Nascimento et al. [25,29] using TG/DTG after adsorption of pyridine, it was possible to quantify the acidity of the catalysts.

Analyzing Table 4, it was observed that the acidity of each material resulted directly from the content of HPMo in the support. Thus, samples with a lower HPM content had a higher density of acid sites, 10HPMo/AlSiM (252 μmol g^−1^) > 20HPMo/AlSiM (215 μmol g^−1^) > 30HPMo/AlSiM (207 μmol g^−1^), which can be attributed to the spreading of HPMo on the AlSiM surface (1071 m^2^ g^−1^) during the anchoring synthesis, making them more accessible and reactive in the presence of the voluminous pyridine molecule. The 10HPMo/AlSiM for presenting higher SSA (907 m^2^ g^−1^) may also have the most accessible acid sites in comparison to the other catalysts in the series, thus presenting higher density of acid sites [28,57].

Although concentrations of acid sites measured by titration with NaOH are very high, there is some inconsistency in the results obtained with pyridine. This can be attributed to steric restrictions on the diffusion of pyridine into the porous system, whereas hydroxyl (OH) appears to detect more active sites than pyridine. As the adsorption of pyridine must occur on cations or hydroxyl groups (Brønsted or Lewis acid sites) located on the external surface or near the entrance of the pores, this hypothetically hinders the access of other pyridine molecules to the free acid sites located inside the pores. This situation was also observed by Campelo et al. [76] in the study of surface acidity with hydroxyl groups (ammonia) and pyridine.

Qualitatively, the FTIR spectra of the adsorbed pyridine samples in the 1700 to 1400 cm^−1^ region can be seen in Figure 4d, with typical bands that can be attributed to the pyridine bound to the Brønsted (B) sites in 1540 and 1637 cm^−1^ and Lewis and Brønsted (B + L) in 1488 cm^−1^. The 1488 cm^−1^ band is enhanced by the presence of M–OH–M sites in which pyridine could bind simultaneously to the H atom (site B) and the metal atoms M (L sites) [28,51].

From the analysis of the FTIR spectra of pyridine adsorption, it can be concluded that these catalysts have Brønsted acid sites. A possible explanation for the observed trend is probably the formation of more reactive MoO_3_ species interacting with silanol groups in AlSiM via dehydration or condensation during calcination favoring the formation of Brønsted acid sites’ Si–O bonds Si–Mo–OH [59,77]. According to the literature Lewis acid site can be transformed into Brønsted acid sites in the presence of water at elevated temperatures [77].

### 3.9. Catalytic Tests

Preliminary investigations on the conversion rates during esterification of eugenol with anhydride was used to probe the catalytic activity of the materials (10, 20–30) HPMo/AlSiM. Control experiments with AlSiM and HPMo were also performed under optimized conditions. As can be seen from Table 5 that AlSiM is not very active for the acetylation of eugenol indicating that the catalytic activity is mainly due to HPMo. The same reaction was carried out by taking the active amount of HPMo (2.5 mg) with 99.9% conversion activity. Almost the same activity was obtained for the (10, 20, 30) HPMo/AlSiM catalyst (20 mg) indicating that HPMo is the actual active species (Table 3). Both heterogeneous (10, 20, 30) HPMo/AlSiM and homogeneous HPMo catalysts contained almost the same amount of protons and therefore exhibited comparable activities. All solid catalysts tested were active compared to homogeneous HPMo with similar conversions.

With respect to turnover frequency (TOF) values, reflecting the catalytic acid strength, the 10HPMo/AlSiM catalyst (2743 min^−1^) was as active as the homogeneous HPMo (2143 min^−1^). This can be explained in the following way the support also plays an important role in modifying the catalytic activity of HPMo [37,59,60,61]. The higher value of SSA (906 m^2^ g^−1^) to 10HPMo/AlSiM, when compared to (20, 30) HPMo/AlSiM, is responsible for the higher catalytic activity. The total acid sites are larger (252 μmol g^−1^) than (20, 30) HPMo/AlSiM, even with the HPMo loading (%) being lower than the others. Therefore, with higher SSA and number acid sites are one of the necessary conditions for catalysis to occur effectively, [37,59] thus 10HPMo/AlSiM exhibits higher catalytic activity in the conversion of eugenol compared to other samples (Table 5).

Considering that increasing the HPMo load by more than 10% did not result in appreciable conversions of eugenol, which is probably due to the partial occlusion of the pores of the catalyst surface (Table 2) with the extra portions of HPMo possibly exceeding the saturation level of the anchorage points of the AlSiM surface, suggesting the possible formation of HPMo multilayers when anchored to AlSiM [37,59,60,61]. In other words, the acidic sites are less accessible and, therefore, less active during the reaction [28,37,57], which is corroborated by the quantification of acid sites measured by the adsorption of pyridine (Table 4).

The difference in catalytic activity in terms of TOF can be interpreted correlating with the textural characteristics and total acidity (Table 2 and Table 4). The increase in HPMo load resulted in a decrease in SSA, Vp and Dp in the same trend. According to Table 5, as the acidity of the catalyst increases, the TOF value also increases 30HPMo/AlSiM (944 min^−1^) < 20HPMo/AlSiM (1423 min^−1^) < 10HPMo/AlSiM (2743 min^−1^). Interestingly, the values of the eugenol conversions on the materials were similar.

Therefore, it should be noted that the TOF values and the conversions to HPMo supported may not truly represent their activity due to the leaching of HPMo with the contribution of the homogeneous catalysis [28,30]. Thus, the leaching of HPMo in the reaction medium could to a certain extent be considered a homogeneous reaction. This shows a clear relationship between the activity of the catalysts (10, 20, 30) HPMo/AlSiM and their leaching: the higher the leaching, the greater the activity [28,30,57].

The leaching of the heteropolianion [PMo_12_O_40_]^3−^ from the catalysts was confirmed by means of a UV-vis analysis based on the absorption at 220 nm and 310 nm from the post reaction mixture [28,69]. Analysis of the post reaction mixture (10HPMo/AlSiM) shows that less than 3% of HPMo (57 μg) was present. In this case, it is believed that the reaction occurred in the predominantly heterogeneous stage, since leaching of the active phase of the catalyst should not exceed 3% [28,49]. While, 20HPMo/AlSiM and 30HPMo/AlSiM were strongly leaching (555 and 1350 μg of HPMo) and possibly their activities had contributions of 15% and 25% of homogeneous catalysis in this reaction. Therefore, it has been found that 10HPMo/AlSiM is more stable for leaching, losing about 3% of [PMo_12_O_40_]^3−^ in the reaction medium.

Therefore, the catalytic activity of 10HPMo AlSiM is functionally related to parameters such as textural properties, SSA, Vp and Dp, and the number of active sites available for catalytic reaction [28,37,57]. Thus, the maximum TOF value was reached with 10HPMo/AlSiM, making it the most active catalyst among the others in the series, and this was named for a more detailed investigation.

### 3.10. Mechanism of Acetylation of Eugenol with Acetic Anhydride

**Scheme 4:**
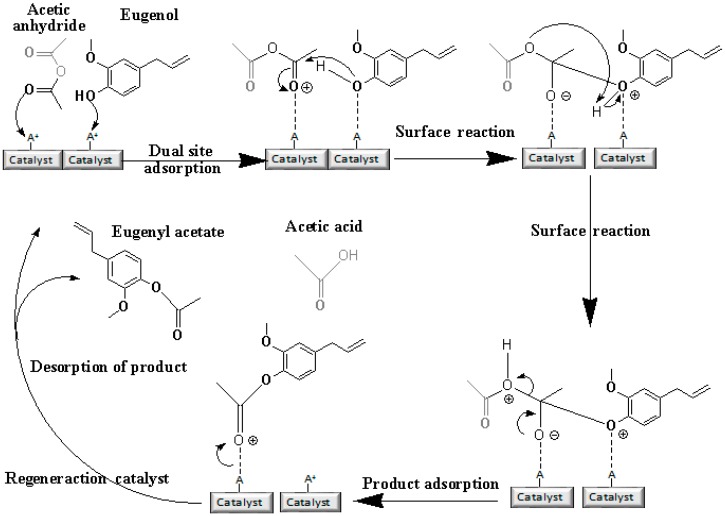
Proposal of mechanism for the reaction of eugenol acetylation on acidic solid catalyst (A^+^ = Brønsted and Lewis acid sites) adaptation [2].

The ester derivative of eugenol was obtained by starting from the synthetic route using acetic anhydride (carboxylic acid) in reactions catalyzed by acidic solids as described in the literature [11,12]. Both eugenol and acetic anhydride are adsorbed at the active sites of the catalysts and the reaction proceeds through the formation of carbocations and the release of acetic acid (Scheme 4). The acylating agent (acetic anhydride) forms a strong electrophile when treated by acid catalysts (Brønsted and Lewis acid sites) that are susceptible to nucleophilic attack (eugenol hydroxyl) [11,12,18].

Thus, the reaction was assumed to occur according to the Langmuir–Hinshelwood–Hougen–Watson (LHHW) mechanism: (i) both reactants (eugenol and acetic anhydride) are adsorbed at the active sites on the catalyst surface; (ii) the reaction occurs between both reagents adsorbed on the surface; (iii) throughout, the process of diffusion is considered relatively fast compared to the surface reaction between adsorbed reagents (Scheme). Considering these premises, the catalyst increases the rate of reaction through its ability to adsorb the reactants such that the activity energy is reduced relative to it without catalyst [2,78].

Under these conditions, the esterification reaction with bulky-chain alcohols (eugenol) follows a LHHW mechanism (Scheme 4), since both eugenol and acetic anhydride have a similar polarity, favoring the adsorption of both reagents at the same active sites on the catalyst surface [79].

First, the reaction starts with the adsorption process where the protons (Al^3+^ or Mo^3+^) generated from the catalyst attract the solitary pair of electrons in the carbonyl oxygen of the acetic anhydride molecule to form an ionic bond; then (reaction) interaction of the reactants on the surface of the catalyst, protonation of the carbonyl of acetic anhydride, occurs; and consequently there is formation of a reactive extremity electrophilic (carbocation) intermediate [80,81].

In the next step (surface reaction), the solitary electron pair (nucleophile) of the oxygen of the eugenol molecule then attacks the electrophile, forming a protonated tetrahedral intermediate. In this tetrahedral intermediate occurs the transference and rearrangement of hydroxyl protons (H^+^) to the carbonyl oxygen (acetic anhydride) that results in the rearranged tetrahedral intermediate formation [2].

In the desorption step of the reaction products in the rearranged tetrahedral intermediate the loss of an acetic acid molecule results in a protonated ester which is still adsorbed to the acid site on the catalyst surface [2].

Thus, with the diffusion of acetic acid into the reaction medium, the desorption of the tetrahedral intermediate (protonated ester) is initiated with the transfer of electrons from the acid site (catalyst) to the oxygen of the carbonyl, thus breaking the bond and simultaneously the deprotonation of the ester and the regeneration of the acid site of the catalyst [2,63].

### 3.11. Reaction Temperature

**Figure 7:**
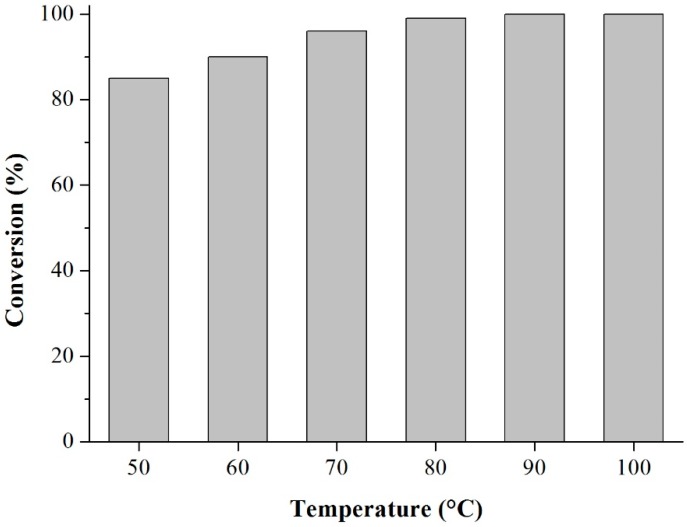
Effect of acetylation reaction temperature. Reaction conditions: EugOH: AA molar ratio (1:5), reaction time 40 min and 2% catalyst.

To evaluate the effect of temperature (50, 60, 70 and 80 °C) it is possible to follow the course of the conversion reaction as shown in Figure 7. The effect of temperature on conversion under similar conditions was studied in the range of 50–100 °C. It can be seen that the conversion increases with the temperature rise from 50 °C (85%) to 70 °C (96%) in that order, and above the conversion rate to 80 °C (99%), above that temperature the conversion is remained unchanged (100 °C was 99.9%). According to Laroque et al. [11] and Santos et al. [18] with the increase of the temperature there is a reduction of the viscosity of the reaction mixture favoring the interaction between active sites of the catalyst and the substrates, thus raising the reaction rate in the acetylation of eugenol. On the one hand, these results are in good agreement with those reported by Tischer et al. [10] using Lewatit^®^ GF 101 and, on the other hand, are better than those obtained with UDCaT-5/Zr [2].

### 3.12. Amount of Catalyst

**Figure 8:**
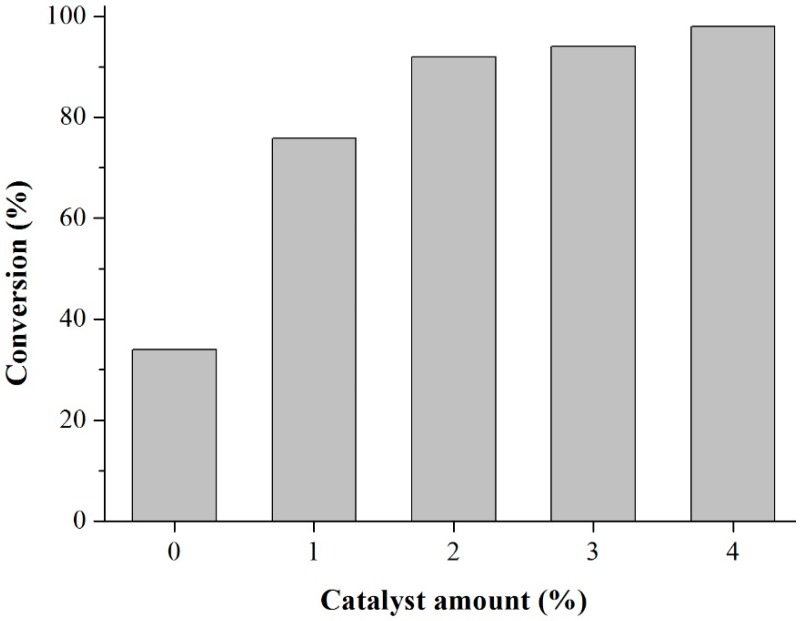
Effect of the amount of catalyst on the acetylation reaction. Reaction conditions: 1:5 molar ratio (EugOH: AA), 80 °C, run time 40 min.

Knowing the mechanisms of the reaction under study, it is known that autocatalysis can occur [4,10], therefore, tests were carried out under the conditions studied without the catalyst and it was observed that the autoconversion did not exceed 35% (Figure 8). This shows that the high performance of the catalyst in the rate of reaction of the reaction is directly proportional to the load of the catalyst based on the total volume of the reaction [2,10,11,82,83]. The catalyst loading was varied from 1% to 4%, relative to eugenol masses, maintaining a 1:5 molar ratio.

It was concluded that both the reaction rate and the conversion rate increased from 74% to 93% with increasing catalytic load (from 1% to 2%, respectively) due to the increase of the available active sites for the reaction [2,11,12]. The aromatic ring of eugenol may be a barrier to access the catalytic sites, necessitating the use of a larger mass of the catalyst. This explanation is corroborated by the results presented in the adsorption of pyridine (Table 3). The reaction rate was almost unchanged for catalyst load of 3% and 4% with conversions of 94% and 99%, respectively. Possibly, this may have occurred because of the fact that above a certain concentration of catalyst the number of active sites was higher than actually required by the substrate [2]. Therefore, the percentage of 2% of catalyst represents the adequate level of active sites to carry out the acetylation of eugenol and the equilibrium of the reaction is reached.

### 3.13. Molar Ratio between Reagents

**Figure 9:**
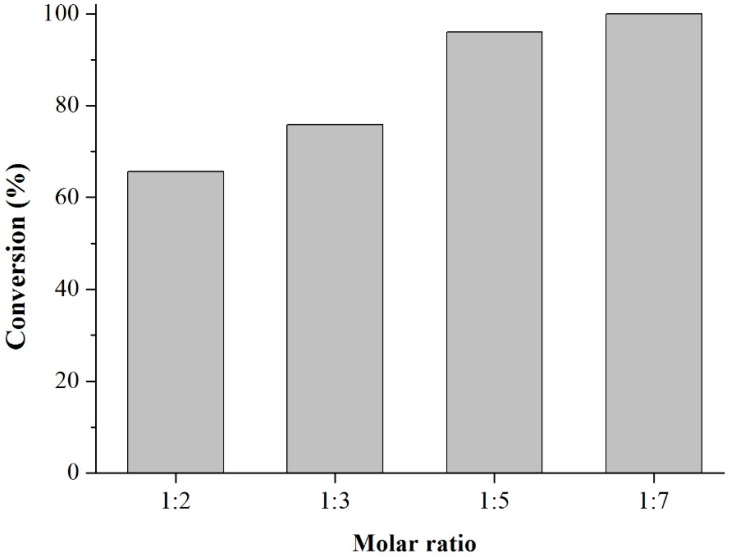
Effect of the molar ratio between the reactants (EugOH and AA). Conditions of the esterification reaction: reaction time 40 min, temperature 80 °C and 2% of catalyst.

The molar ratio between the substrates is generally one of the most important parameters in the esterification reactions. As the reaction is reversible, an increase in the concentration of a substrate (particularly, acetic anhydride) may displace the chemical balance, resulting in higher conversions [1,11,12]. In this study, higher conversions were obtained using the 1:5 molar ratio, however in 40 min high conversions were obtained (96%) (Figure 9). It was observed that, even increasing the molar ratio of 1:5 to 1:7, no significant increase in conversion (99%) was observed on the catalyst. Thus, the main positive effect in the conversion was the excess acetic anhydride/eugenol molar ratio as reported in the literature [18]. Thus, all subsequent reactions were conducted at a molar ratio of 1:5 eugenol with acetic anhydride.

### 3.14. Reaction Time

**Figure 10:**
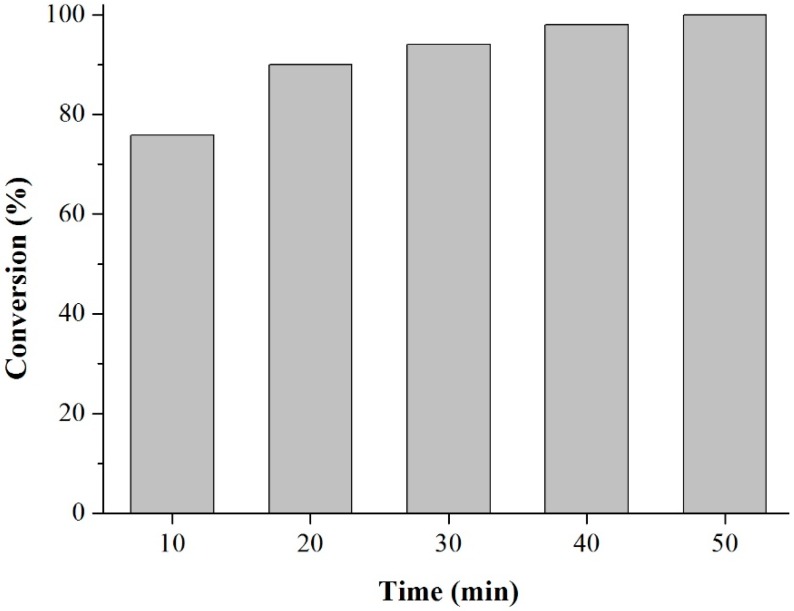
Effect of time on acetylation reaction. Reaction conditions: 1:5 molar ratio (EugOH: AA), 80 °C and 2% catalyst.

The effect of the reaction time on the conversion of eugenol was investigated. The reaction time is the most important parameter to obtain high conversion values of the product of interest [2,4,10,11]. As expected for esterification reaction, conversion rates ranged from 75% (10 min) to 98% (40 min), the highest conversion obtained was 99.9% after 50 min (Figure 10). It was observed that the value of the reaction rate was higher in the intervals of 10 to 30 min with conversions of 75% to 96% respectively, while from 30 to 50 min the conversion was 96% to 99.9%. According to Machado et al. [4] the increase in ester conversion is due to the prolongation of the contact time between the substrate and the catalyst, which increases the mass transfer and consequently leads to higher conversions. The results obtained here after 40 min of reaction are better than those obtained by Laroque et al. [11] using Molecular sieve 4 Å to catalyze the conversion of eugenol after 2 h.

Therefore, optimized conditions for acetylation of eugenol over 10HPMo/AlSiM are: molar ratio for 1:5 (eugenol: acetic anhydride); amount of catalyst 2% (*w*/*w*, EugOH); temperature and reaction time were 80 °C and 40 min respectively.

The excellent catalytic performance of the present catalysts lies in obtaining a higher conversion of eugenol under reaction conditions similar to those reported in Table 6, where the kinetic parameters served as the basis for our discussion. The results show that the maximum eugenyl acetate conversions obtained in this study (>99.9%) were higher than many values found in the literature (Table 6).

It can be seen from Table 6 that conversions of 66% [20] and 93% [1] were obtained using biocatalysts, although the molar ratio and the reaction temperature were minimized, but the disadvantages of the biocatalyst are well emphasized as low reuse efficiency and high reaction times make the process costly [1,10]. The UDCaT-5 [2] shows considerably high conversions, but high reaction time and temperature were used. *Amberlyst A-21* [12] and 4 Å molecular sieves [11] catalysts also require prolonged reaction times. Also, the recovery of these catalysts requires the use of organic solvents, hexane and toluene. It is noticed that, although the conversions obtained in these works were high, the reaction times in the majority of cases were much superior in comparison to the reaction times realized in this work.

Therefore, the catalyst developed in this work seems to be a promising alternative, since it is obtained from inexpensive, easy to handle raw material, excellent acetylating agent and environmentally friendly for a sustainable production of eugenol acetate, a product of great commercial value.

### 3.15. Kinetic Study of the Reaction

**Figure 11:**
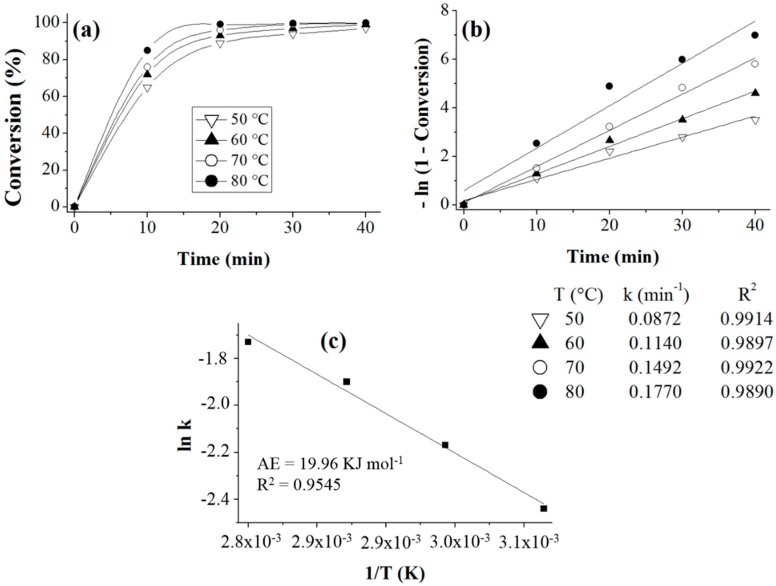
Kinetic study of the conversion of eugenol to the catalyst (**a**); kinetic plot of −ln (1 − conversion) versus time at different temperatures for calculations of velocity constants and reaction order analysis, where (**b**); Arrhenius graph for calculating the activation energy for eugenol acetylation reaction at different temperatures on the catalyst (**c**).

The kinetic study was performed based on the best experiments of the previous sections. In order to evaluate the effect of temperature (50, 60, 70 and 80 °C) and time (10, 20, 30 and 40 min) on the eugenol conversions, obtaining the conversion curves as a function of time, we can see the effect of varying the temperature in Figure 11a. From the kinetics shown in Figure 11a, it was possible to observe the high performance of 10HPMo/AlSiM in the reaction. According to Santos et al. [18] and Machado et al. [4] the temperature increase may improve the diffusion of the substrates or their solubility over the reaction time, as well as the maximum reaction rate which increased from 50 to 80 °C, suggesting a positive effect of the temperature on the rate of esterification. As shown in Figure 11a, high conversions were obtained within the first 20 min of reaction (90, 93, 96 and 98%, respectively), and after 40 min there was a slight increase in reaction rates (96, 98, 99 and 99.9%).

According to the analyzed kinetic data obtained experimentally on the catalyst (Figure 11a), with acetic anhydride in excess in molar ratio to eugenol, the reaction becomes kinetically of the pseudo first order [2,11]. Thus a graph of −ln (1 − conversion) versus time was plotted at different temperatures (Figure 11b). Thus, using graphical procedures, it was concluded that the kinetic data analyzed fit well with first order kinetics in relation to eugenol, since they generated linear regression coefficients (R^2^ > 0.98) very close to the unit for the reactions performed at 50, 60, 70 and 80 °C, respectively (Figure 11b). Therefore, the acetylation of eugenol with acetic anhydride is considered to have followed a first order kinetics, as described in the literature [2,11].

From the linearization of −ln (1 − conversion) by time, the constants (k) of the pseudo first order rate for each temperature were studied, which are shown in Figure 11. With the values of k, the Arrhenius plot was obtained from the linearization of lnk versus 1/T (Figure 11c), obtaining the apparent activation energy (EA) of 19.96 KJ mol^−1^ with good regression linear (R^2^ = 0.9845).

The observed apparent energetic activation value (19.96 KJ mol^−1^) for the esterification of eugenol with acetic anhydride over 10HPMo/AlSiM compared to other works, is close to the activation energy (21.40 KJ mol^−1^) obtained by the enzymatic catalysis [18]. The value obtained was lower compared to the activation energy (39.13 KJ mol^−1^) found in the esterification of eugenol with benzoic acid (toluene as solvent) on UDCaT-5 [2]. In addition, comparing with other solid catalysts such as the 4 Å molecular sieve (10.03 KJ mol^−1^) and Amberlite XAD-16 (7.23 KJ mol^−1^) reported by Laroque et al. [11] during the acetylation of eugenol with acetic anhydride, the present catalyst exhibits higher activation energy, however, as far as the reaction condition is concerned, the present catalyst was more active in a shorter reaction time.

### 3.16. Reuse of the Catalyst

**Figure 12:**
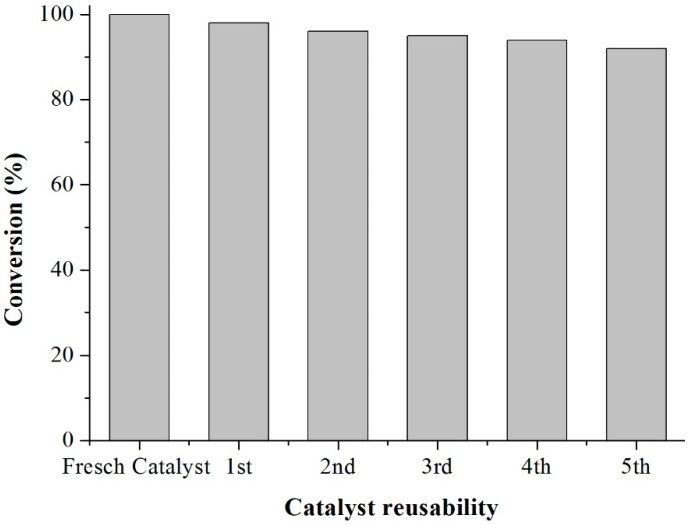
Reusability the 10HPMo/AlSiM catalyst.

The regeneration of the catalyst after 40 min of reaction was performed by centrifugation (6000 rpm for 10 min), washing with ethanol followed by drying at 150 °C for 2 h in an oven and was used in the subsequent esterification reaction with a new reaction mixture. The profile of the reused catalyst can be seen in Figure 12. It was found that the catalyst is still active after subsequent cycles of reuse. The original conversion was not achieved in any of the reuse runs. The conversion of 99.9% of the fresh catalyst falls to 97% in the first run and decreased to about 96% in the 2nd, 94% in the 3rd, 92% in the 4th and 90% in the 5th run, respectively. TOF values also followed a similar trend (Table 7).

This decrease in catalytic activity was attributed to the leaching of HPMo (from about 1 to 3% in each cycle (Table 7) of the support for the liquid phase during the catalytic reactions, which was confirmed by the UV-vis technique [28,51]. The solubilization of the active phase transforms into a mixed system, a homogeneous part and a heterogeneous part. This fact was also observed by Pires et al. [30], and second Lacerda Júnior et al. [49] and Oliveira et al. [28]. HPA leaching is acceptable up to 2.7%.

**Figure 13:**
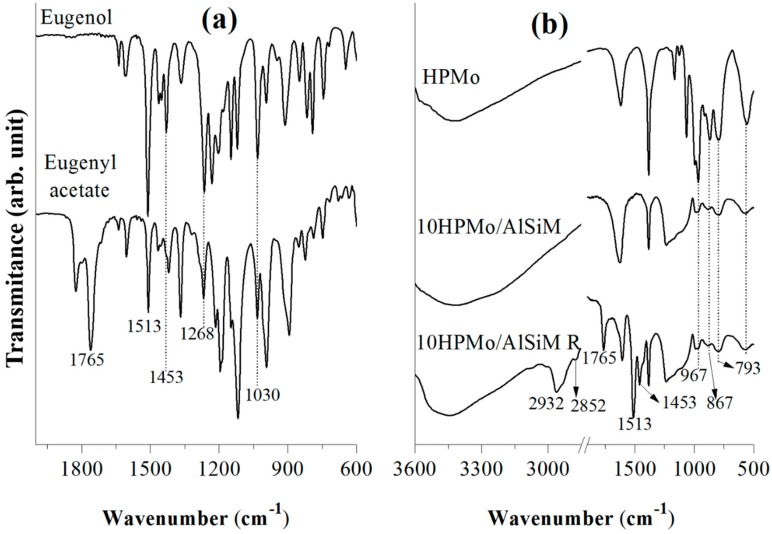
FTIR spectra for eugenol, eugenyl acetate (**a**) and 10HPMo/AlSiM and 10HPMo/AlSiM R (where R = reused) (**b**).

Although the catalyst was washed after centrifugation to remove all adsorbed reagents and products, the possibility of retention of some of the adsorbed reagents and product species still remained (Figure 13b), which may cause blocking of active sites and cause a decrease in catalytic activity. This fact can be attributed to the deactivation of the catalyst is the loss of mass during the friction in the reactor during the reaction and in the recovery process (Table 7) [28,59]. Even so, the conversion remained higher than that of autocatalysis (>35%), that is, >90%. Therefore, 10HPMo/AlSiM catalyst showed capacity with application of reuse expressed by TOF, making feasible and environmentally sustainable the use of this material in the esterification of eugenol. This also confirms the truly heterogeneous, as well as active and reusable mode of action.

### 3.17. Characterization of Reaction Components

The identification of the reaction components obtained in this work were performed by FTIR and CG/MS. Following the FTIR spectra (Figure 13a) shows characteristic vibrational bands of eugenol and eugenol acetate. In the eugenol spectrum, bands at 3468 cm^−1^ (relative to the stretching of the O–H bond of the hydroxyl group) are observed at 1268 cm^−1^ (stretching of the C–O bond of the hydroxyl-bound carbon) and at 1513 and 1030 cm^−1^ both referring to the aromatic ring and aromatic ether respectively. In the FTIR spectrum of the eugenol acetate analysis, the presence of the characteristic carbonyl ester band attached to the aromatic ring is observed at 1765 cm^−1^, and at 1453 cm^−1^ is attributed to the methyl group, confirming that only the acyl group was added for the eugenol molecule. The spectra obtained in this study were similar to those found by Affonso et al. [84] and Machado et al. [4].

Regarding the GC-MS analysis, comparing the data obtained for eugenol and eugenil acetate samples, it was observed that the retention time (RT) values and retention indices (RI) were very similar (24.9 min and 1376 for eugenol and 31.6 min 1535 for eugenyl acetate, respectively) (Table 8). The retention indices reported in the literature for these compounds are about 1350 (eugenol) and 1513 (eugenyl acetate) [54,55]. The set of these results and the evaluation of the chromatography profiles are shown in Figures S1 and S2 which prove that eugenol and eugenil acetate are the components present after the reactions.

### 3.18. Characterization of the Reused Catalyst

In order to verify the integrity of the catalyst after successive esterification reactions, after the fifth reuse cycle the same was characterized by FTIR, DRS and XRD. First, the FTIR analysis was undertaken and the catalyst spectra before and after the reuse are shown in Figure 13b. In the FTIR spectrum of the 10HPMo/AlSiM material prior to the first esterification cycle a broad band between about 3600–3200 cm^−1^ relative to OH is observed, suggesting the presence of water, which is confirmed by the band at 1633 cm^−1^ [28,59]. There were no bands related to organic molecules that confirmed the presence of ester. However, the spectrum for the catalyst used (10HPMo/AlSiM R) (Figure 13b) presented some new prominent adsorption bands near 2932 and 1453 cm^−1^, attributed to the symmetrical and asymmetric stretching of methyl (CH_3_), 1765 cm^−1^, attributed to the ester carbonyl (CO) stretch bond, 1513 cm^−1^, referring to the presence of an aromatic ring [4,84] which demonstrates the binding of the esterification product on the surface of the reused catalyst. Therefore, these bands are attributed to adsorption characteristics of ester molecules on the surface of the catalyst that can cause reduction in catalytic activity when it is reused [28]. It is interesting to note that even after 10HPMo/AlSiM R was used in the catalytic reactions, the main HPMo absorption peaks were observed in the range of 967, 867 and 793 cm^−1^ for Mo–O and Mo–O–Mo respectively [28,85].

**Figure 14:**
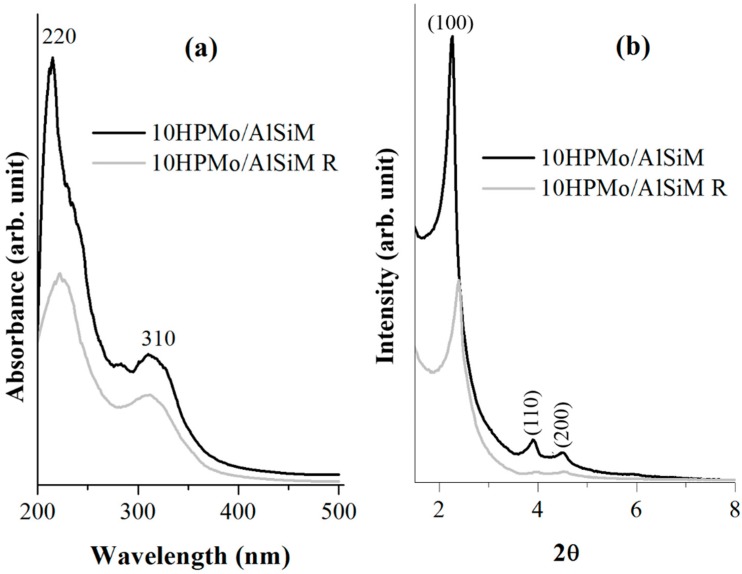
Data of fresh and reused catalysts: (**a**) Comparison of the DRS spectra; (**b**) XRD patterns.

To see any change in the catalyst structure after the completion of the reaction, the DRS of the catalyst used was analyzed. Figure 14a illustrates the DRS spectra of the fresh catalyst as well as the catalyst used. A small difference in intensity at the 220 and 310 nm peaks of the DRS is observed indicating the stability of the catalyst used after the five reaction cycles [61,68,69,70]. In addition, the filtrate of the reaction mixture was also checked for the presence of any HPMo leached by UV-vis (Table 7). The low concentration (<3%) of the heteropolyion [PMo_12_O_40_]^3−^ in the reaction solution indicates that there is no appreciable leaching of HPMo [28]. All FTIR and DRS spectra suggest that the Keggin structure (HPMo) remains intact in the support even after the reaction [28,72,85].

XRD patterns (Figure 14b) are almost identical when new and used, indicating that the hexagonal symmetries as can be seen are still preserved [30,61]. What is clearly seen is only a decrease in the reflection intensity (100) as well as the partial disappearance of the reflections (110) and (200) for the spent catalyst which may indicate the collapse of the structure. This effect may be due to the adsorption of molecules on the surface and within the channels (Figure 13b) which consequently indicates a structural distortion of the support [61]. This result is promising given the known fragility of these types of mesoporous systems with respect to stability under reaction conditions [61].

Considering other studies of catalysts reporting toluene [2] and hexane [10,11] with very high reaction times, the superiority of the present catalyst, prepared in this work, is to obtain a greater conversion of eugenol under moderate reaction conditions, including minimum leaching of the active species of HPMo, good recyclability with moderate loss of activity and simple regeneration.

## 4. Conclusions

*Flint* kaolin, a wasted kaolinite was used for the first time as a support for catalytic esterification of eugenol. The reaction of eugenol with acetic anhydride was carried out on a series of solid (10%, 20% and 30%) HPMo acid catalysts anchored in AlSiM prepared from this waste kaolin. The influence of different reactional parameters was investigated and revealed that 10HPMo/AlSiM at 8.5 wt. % actual HPMo had the highest leaching stability and catalytic activity during the above reaction, achieving 99.9% eugenol conversion. The characterization results of XRD and FTIR and DRS obtained for the 10HPMo/AlSiM catalyst confirmed the preservation of HPMo before and after the reaction. Therefore, HPMo Keggin anions were the active species on the surface of the catalyst. The catalytic performance of 10HPMo/AlSiM should be attributed to the high surface acidity (252 μmol g^−1^ of Py) of the material, high SSA (906 m^2^ g^−1^), Vp (0.77 cm^3^ g^−1^) and its large size (3.7 nm) which facilitated the diffusion of the substrate and the product. 10HPMo/AlSiM showed no loss of activity and conversion over 90% was ensured for five cycles of reuse. Notable features of this method are its simplicity of operation, short reaction time, cleaner reaction processes, low cost, high catalyst stability and reusability, and excellent conversion of eugenol. Therefore, a completely new and heterogeneous green process was obtained for the synthesis of a molecule with potential larvicidal activity.

## Figures and Tables

**Table 1:** Chemical composition of precursors, AlSiM and (10, 20 and 30) HPMo/AlSiM through X-ray fluorescence (XRF).

Samples	SiO_2_	Al_2_O_3_	TiO_2_	Fe_2_O_3_	P_2_O_5_	MoO_3_	PF	Si/Al	Si/Mo ^a^	Si/Mo ^b^
**KF**	42.3	38.3	3.2	2.9			13.3	1		
**MF**	49.6	37.9	2.5	2.8			1.3	1		
**MFL**	74.0	4.7	3.7	1.6			16,0	13		
**AlSiM**	86.7	3.2	3.5	1.1			5.5	23		
**10HPMo/AlSiM**	74.2	2.8	2.0	0.6	0.5	7.4	12.5	23	20	24
**20HPMo/AlSiM**	63.2	1.4	1.5	0.3	1.0	13.5	19.1	37	9	11
**30HPMo/AlSiM**	54.1	1.4	0.7	0.5	0.6	15.7	27.0	33	5	8

^a^ Theoretical calculation for Si/Mo present in the support before washing. ^b^ Calculation of Si/Mo after washing.

**Table 2:** Structural and textural properties of AlSiM and (10, 20 and 30) HPMo/AlSiM.

Samples	d*_100_* (nm) ^a^	a_0_ (nm) ^b^	w_t_ (nm) ^c^	SSA (m^2^ g^−1^) ^d^	SS_p_ (m^2^ g^−1^) ^e^	V_p_ (cm^3^ g^−1^) ^f^	D_p_ (nm) ^g^
**KF**				8 ± 0.2	5.7	0.05	32.20
**MF**				9 ± 0.2	5.9	0.05	31.11
**MFL**				433 ± 6.6	127.5	0.56	1.20
**AlSiM**	4.35	5.02	1.17	1071 ± 6.5	1376.6	1.05	3.85
**10HPMo/AlSiM**	4.27	4.93	1.22	906 ± 6.4	1305.5	0.77	3.71
**20HPMo/AlSiM**	3.95	4.56	1.33	869 ± 6.4	1274.3	0.65	3.23
**30HPMo/AlSiM**	3.98	4.60	1.39	728 ± 6.4	909.5	0.57	3.21

^a^ d*_100_* = Baseline reflection interplanar distance (d*_100_* = λCuKα/senθ). ^b^ a_0_ = Network parameter for a hexagonal structure (2d*_100_*/√3). ^c^ W_t_ = Thickness of pore wall (W_t_ = a_0_ − D_P_). ^d^ SSA = Specific surface area calculated by the Brunauer-Emmett-Teller (BET) method. ^e^ SSp = Surface area of pores calculated by the BET method. ^f^ V_P_ = Volume of pores calculated by the Barret-Joyner-Halenda (BJH) method. ^g^ D_P_ = Pore diameter calculated by the BJH method.

**Table 3:** Synthesis of mesoporous aluminosilicate from kaolinite precursors.

Materials	Precursors	SSA (m^2^g^−1^)	V_p_ (cm^3^g^−1^)	D_p_ (nm)	Ref.
**Al-MCM-41**	Metakaolin	877	0.78	2.76	[44]
**Al-MCM-41**	Metakaolin	1016	0.80	3.14	[27]
**Al-MCM-41**	Kaolin	1041	0.97	3.7	[50]
**M2C1A1**	Kaolinite	1420	1.37	2.78	[45]
**Al-MCM-41**	Metakaolin	753	0.63	3.33	[46]
**AlSiM**	Kaolin *flint*	1071	1.05	3.85	Present work

**Table 4:** Evaluation of the number of acid sites in catalyst materials.

Samples	Number of Acid Sites (mmol H^+^ g^−1^) ^a^	Number of Acid Sites (μmol g^−1^) ^b^
**HPMo**	20.74	
**AlSiM**	1.31	
**10HPMo/AlSiM**	4.38	251.85
**20HPMo/AlSiM**	5.92	215.23
**30HPMo/AlSiM**	7.22	207.65

^a^ Total surface acidity of the actual catalysts calculated by titration [26,28,30]. ^b^ Number of mole of pyridine calculated by TGA/DTG analysis [25,29]. HPMo = 2.4 mg used in the base acid titration.

**Table 5:** Conversion data (%) and turnover frequency (TOF) for the catalysts (10, 20 and 30) HPMo/AlSiM.

Samples	Catalyst (mg) ^a^	HPMo (mg) ^b^	HPMo (μg) ^c^	Conv. (%) ^d^	TOF (min^−1^) ^e^
**HPMo**		2.5		99.9	2167
**AlSiM**	20			34.7	
**10HPMo/AlSiM**	20	1.9	57	98.5	2743
**20HPMo/AlSiM**	20	3.7	555	99.2	1423
**30HPMo/AlSiM**	20	5.4	1350	99.4	944

^a^ Mass of catalyst (10, 20, 30) HPMo/AlSiM used in the reaction. ^b^ Mass of HPMo corresponding to the amount (x%) present in AlSiM. ^c^ Mass of HPMo leached in the reaction solution, detected by UV-vis. ^d^ Conversion of Eugenol (EugOH: AA molar ratio = 1:5, 2% catalyst, 80 °C, 40 min). ^e^ TOF = (turnover frequency) is defined as the number of moles of eugenol converted per moles of active Mo species in the catalyst in unit time, heterogeneous catalysts assuming that all acid protons are equally active [28,59].

**Table 6:** Comparison of eugenol conversion with reported catalyst.

Catalyst	Solvent	T (°C)	R: M	*T* (min)	CONV. (%)	Ref.
**UDCaT-5/Zr**	Toluene	110	1:5	240	90	[2]
**Amberlite XAD-16**	Acetic anhydride	55	1:3	120	98	[11]
**Molecular sieve 4 Å**	Acetic anhydride	60	1:3	120	90	[11]
**Lipase (RML)**	Chloroform	50	1:3	300	66	[20]
**Lipozyme TL**	Acetic anhydride	70	1:5	120	93	[1]
**Amberlyst A-21**	Acetic anhydride	95	1:3	120	95	[12]
**Lewatit^®^ GF 101**	Acetic anhydride	70	1:1	45	100	[10]
**10HPMo/AlSiM**	Acetic anhydride	80	1:5	40	99.9	Present work

**Table 7:** Leaching of the catalyst after esterification reaction.

Cycles	(mg) ^a^	(mg) ^b^	(μg) ^c^	(%) ^d^	TOF (min^−1^)
**Fresh**	20.04	1.9	54	2.8	3187
**1**	19.10	1.85	24	1.3	3176
**2**	18.05	1.83	20	1.1	2750
**3**	17.20	1.80	20	1.2	2740
**4**	16.15	1.78	30	1.7	2675
**5**	15.35	1.75	35	2.3	2662

^a^ Mass of catalyst recovered after each reuse cycle. ^b^ Mass of HPMo anchored in AlSiM. ^c^ Mass of HPMo leached during the reaction. ^d^ Percentage of leaching in the reaction medium.

**Table 8:** Chemical characterization of eugenol and eugenyl acetate by gas chromatography-mass spectrometry (GC-MS).

Compounds	RT	RI	RI ^a^	Fragmentation Profiles (*m/z*)
Eugenol	24.9	1376	1350	164, 149, 131, 121, 103, 91, 77, 65, 55, 41
Eugenyl acetate	31.6	1535	1513	206, 164, 149, 131, 121, 103, 91, 77, 65, 55, 43

Retention time, RT (minutes), calculated RI, retention index, mass m/z for charge rate. ^a^ RI found in the literature [54,55].

## Supplementary Materials

The following are available online at https://www.mdpi.com/1996-1944/12/18/2995/s1, Figure S1: Mass spectrometry (GC/MS) (**a**) eugenol, (**b**) eugenol and eugenol acetate, (**c**) acetate eugenol, Figure S2: Chromatograms (**a**) eugenol, (**b**) eugenol and eugenol acetate (autocatalysis), (**c**) eugenol and eugenol acetate (catalyzed with 10HPMo/AlSiM), (**d**) eugenol acetate (catalyzed with 10HPMo/AlSiM).
