# Supplementary material for: Acetylation of Eugenol over 12-Molybdophosphoric Acid Anchored in Mesoporous Silicate Support Synthesized from Flint Kaolin
Source: Materials (Basel). 2019 Sep 16;12(18):2995. doi: 10.3390/ma12182995 (PMC6766331; doi:10.3390/ma12182995)
Supplement: Supplementary file 1 [file materials-12-02995-s001.pdf]

materials

MDPI

Supplementary Material

Acetylation of Eugenol over 12-Molybdophosphoric Acid Anchored in Mesoporous Silicate Support Synthesized from *Flint* Kaolin

Alex de Nazaré de Oliveira [1,2,3], Erika Tallyta Leite Lima [1,2], Deborah Terra de Oliveira [2,4], Rômulo Simões Angélica [5], Eloisa Helena de Aguiar Andrade [1,6], Geraldo Narciso da Rocha Filho [1,2], Carlos Emmerson Ferreira da Costa [1,2], Fabiola Fernandes Costa [7], Rafael Luque [8,9] and Luís Adriano Santos do Nascimento [1,2,4,*]

1 Graduation Program in Chemistry, Federal University of Pará, Augusto Corrêa Street, Guamá, Belém PA 66075-110, Brazil
2 Laboratory of Oils of the Amazon, Federal University of Pará, Perimetral Avenue, Guamá, Belém PA 66075-750, Brazil
3 Department of Exact and Technologic Sciences, Federal University of Amapá, Rod. Juscelino Kubitschek, km 02-Jardim Marco Zero, Macapá AP 68903-419, Brazil
4 Graduation Program in Biotechnology, Federal University of Pará, Augusto Corrêa Street, Guamá, Belém PA 66075-110, Brazil
5 Laboratory of X-Ray Diffraction, Federal University of Pará, Augusto Corrêa Street, Guamá, Belém PA 66075-110, Brazil; rsangelica@gmail.com
6 Adolpho Ducke Laboratory, Botany Coordinating, Museu Paraense Emílio Goeldi, Perimetral Avenue, Terra Firme, Belém PA 66077-830, Brazil
7 Campus of Salinópolis, Federal University of Pará, Salinópolis, Pará CEP 68721-000, Brazil
8 Faculty of Sciences, Research Institute of Chemistry, Peoples Friendship University of Russia (RUDN University), 6 Miklukho Maklaya str., 117198 Moscow, Russia; q62alsor@uco.es
9 Department of Organic Chemistry, Universidad de Córdoba, Ctra Nnal IV-A, Km 396, E14014 Cordoba, Spain
* Correspondence: adrlui1@yahoo.com.br; Tel.: +55-91-98171-4947

Received: 15 July 2019; Accepted: 9 September 2019; Published: 16 September 2019

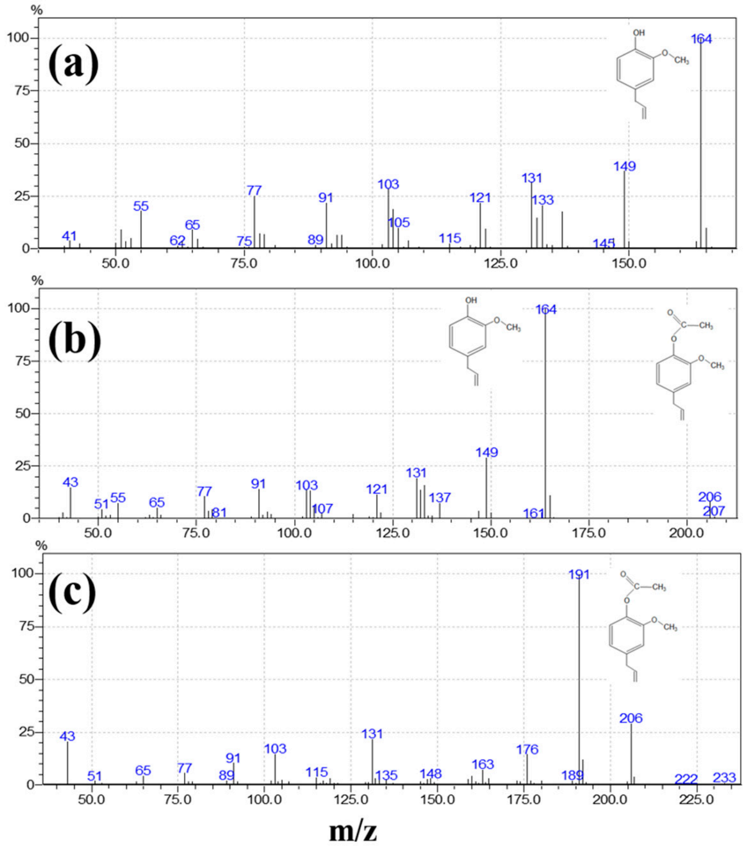

Figure S1. Mass spectrometry (GC/MS) (**a**) eugenol, (**b**) eugenol and eugenol acetate, (**c**) acetate eugenol.

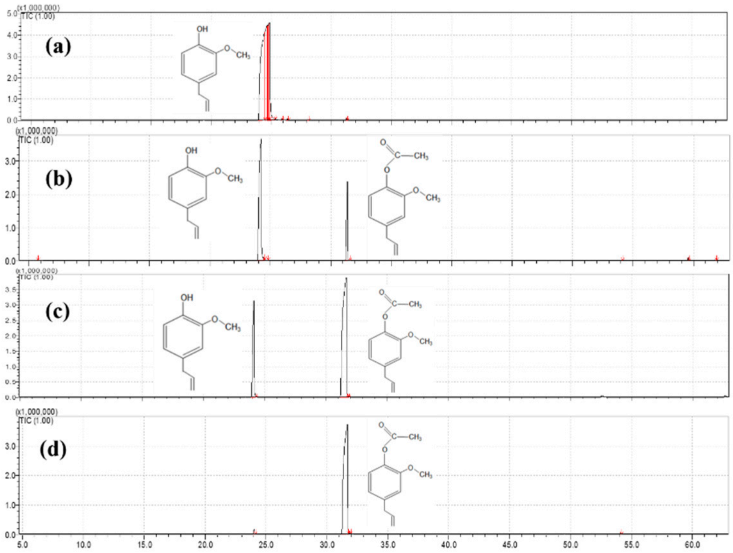

Figure S2. Chromatograms (**a**) eugenol, (**b**) eugenol and eugenol acetate (autocatalysis), (**c**) eugenol and eugenol acetate (catalyzed with 10HPMo/AlSiM), (**d**) eugenol acetate (catalyzed with 10HPMo/AlSiM).

© 2019 by the authors. Licensee MDPI, Basel, Switzerland. This article is an open access article distributed under the terms and conditions of the Creative Commons Attribution (CC BY) license (http://creativecommons.org/licenses/by/4.0/).
